# Microheater Controlled Crystal Phase Engineering of Nanowires Using In Situ Transmission Electron Microscopy

**DOI:** 10.1002/smtd.202400728

**Published:** 2024-09-23

**Authors:** Christopher R. Y. Andersen, Marcus Tornberg, Sebastian Lehmann, Daniel Jacobsson, Kimberly A. Dick, Kristian S. Mølhave

**Affiliations:** ^1^ DTU Nanolab Technical University of Denmark Kgs. Lyngby 2800 Denmark; ^2^ Centre for Analysis and Synthesis NanoLund Lund University Box 124 Lund S‐221 00 Sweden; ^3^ DTU Electro Technical University of Denmark Kgs. Lyngby 2800 Denmark; ^4^ Quantum DTU Technical University of Denmark Kgs. Lyngby 2800 Denmark; ^5^ Solid State Physics NanoLund Lund University Box 118 Lund S‐221 00 Sweden; ^6^ nCHREM Lund University Box 124 Lund S‐221 00 Sweden

**Keywords:** crystal phase engineering, epitaxy, in situ, MEMS, nanowires, TEM, temperature

## Abstract

Crystal Phase Quantum Dots (CPQDs) offer promising properties for quantum communication. How CPQDs can be formed in Au‐catalyzed GaAs nanowires using different precursor flows and temperatures by in situ environmental transmission electron microscopy (ETEM) experiments is studied. A III‐V gas supply system controls the precursor flow and custom‐built micro electro‐mechanical system (MEMS) chips with monocrystalline Si‐cantilevers are used for temperature control, forming a micrometer‐scale metal–organic vapor phase epitaxy (µMOVPE) system. The preferentially formed crystal phases are mapped at different precursor flows and temperatures to determine optimal growth parameters for either crystal phase. To control the position and length of CPQDs, the time scale for crystal phase change is investigated. The micrometer size of the cantilevers allows temperature shifts of more than 100 °C within 0.1 s at the nanowire growth temperature, which can be much faster than the growth time for a single lattice layer. For controlling the crystal phase, the temperature change is found to be superior to precursor flow, which takes tens of seconds for the crystal phase formation to react. This µMOVPE approach may ultimately provide faster temperature control than bulk MOVPE systems and hence enable engineering sequences of CPQDs with quantum dot lengths and positions defined with atomic precision.

## Graphical abstract

Crystal phase quantum dots (CPQDs) in GaAs nanowires are precisely controlled using resistively heated cantilever chips in an environmental transmission electron microscope. This work demonstrates that temperature adjustments, rather than precursor flow, dictate the crystal phase better in opposition to other studies, enabling rapid and precise CPQD engineering with atomic precision in position and length offering potential for advanced quantum technologies.

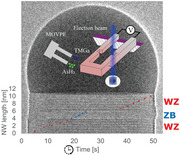

## 1 Introduction

Crystal phase quantum dots (CPQDs) in III‐V nanowires have shown to be a promising platform for optical and electrical applications within nanophotonics and quantum technologies.^[^ ^1^, ^2^, ^3^ ^]^ Non‐nitride III‐V nanowire‐based quantum dots are of particular interest for this reason individual CPQDs have been studied for the last decade.^[^ ^4^, ^5^, ^6^, ^7^, ^8^ ^]^ Despite prominent results for single quantum dots, multiple CPQDs, and their mutual interactions are still to be investigated experimentally.^[^ ^9^ ^]^ This calls for a deeper understanding and a high quality of crystal phase engineering to reach a precise size and position control of the target structure. CPQDs rely on a change in crystal structure rather than composition within the heterostructure.^[^ ^10^, ^11^, ^12^ ^]^ This provides multiple experimental advantages such as; 1) atomically sharp interfaces can be formed without the risk of compositional intermixing, 2) reduced strain across the interface,^[^ ^13^ ^]^ 3) precise control of the QD excitation energy stemming from atomically sharp interfaces and size control, and 4) well‐defined precision in positioning multiple quantum dots with respect to each other.

Crystal phase control, namely the switching between wurtzite (WZ) and zincblende (ZB), has experimentally been achieved by tuning the growth conditions such as temperature^[^ ^14^, ^15^, ^16^, ^17^ ^]^ and/or precursor flow.^[^ ^15^, ^16^, ^18^, ^19^, ^20^ ^]^ Engineering of the crystal phase formation by only varying the group V‐flow has been pointed out as a versatile approach for ex situ growth due to its simplicity.^[^ ^18^, ^19^ ^]^ Temperature has also been used successfully in ex situ growth chambers for crystal phase engineering, but it is challenging to ultimately control, more time‐consuming to change, and growth temperature is a complex parameter for the theoretical description of the crystal growth.^[^ ^14^, ^17^, ^18^ ^]^ So far, the reports dedicated to controlling CPQD formation are exclusively based on ex situ studies, but the lack of monitoring of the exact crystal layers, as they form, makes it difficult to control and correlate the positions of multiple quantum dots. Recently, in situ, transmission electron microscopy with integrated metal–organic vapor phase epitaxy (MOVPE) or molecular beam epitaxy (MBE) capabilities has resulted in important findings when studying the control of the crystal phase formation as it happens. This approach has been used for GaAs nanowires, in particular, to map out the crystal phase formation,^[^ ^21^, ^22^ ^]^ growth rate,^[^ ^23^, ^24^, ^25^ ^]^ and size dependency^[^ ^26^ ^]^ at different precursor flows. The role of temperature on crystal phase formation and nanowire growth is still to be investigated more systematically in situ.

In this Research Paper, we investigate, in situ, the CPQD formation in Au‐catalyzed GaAs nanowires. Dominating crystal phases are mapped at different precursor flows and temperatures to determine optimal growth parameters for either crystal phase, WZ, or ZB. To control the position and length of the CPQDs, the time required for the crystal phase change is investigated when varying either the precursor flow or the temperature. The precursor control and associated growth observations are achieved by using an environmental transmission electron microscope (ETEM) with access to a III‐V gas‐handling system.^[^ ^27^ ^]^ Temperature is controlled by custom‐built and calibrated micro electro‐mechanical system (MEMS) chips with resistively heated micrometer‐sized Si cantilevers, and microheaters, forming what could be called a µMOVPE system.^[^ ^28^, ^29^ ^]^ This µMOVPE approach ultimately provided access to in situ observations not possible in bulk MOVPE systems and hence enables engineering routines for sequences of CPQDs with atomic precision of lengths and positioning.

## 2 Results and Discussion

**Figure 1:**
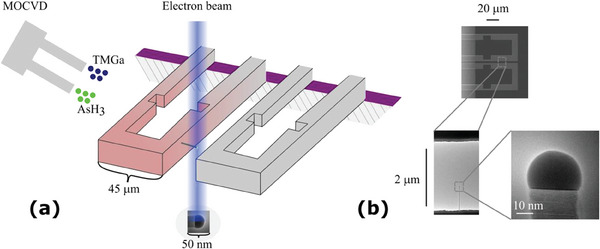
Schematic a) of two neighboring microheaters on a chip mounted in the ETEM. The illumination of a nanowire growing epitaxially from the microheater sidewall is indicated by the resulting image of the nanowire. The precursors are injected from two separate side ports into the microscope column. The resulting ex situ SEM and in situ TEM images b) illustrate the microheaters and low and high magnification of a nanowire, respectively.

Au‐catalyzed GaAs nanowires were monitored while growing in an ETEM with an integrated MOVPE system supplying trimethylgallium (TMGa) and arsine (AsH_3_). Controlled by the supply of growth species and temperature, epitaxial nanowire nucleation and growth on the silicon (111) cantilever microheater sidewalls occurred as illustrated by the schematic in **Figure** **1**. The precursor flows of the growth species were adjusted by mass flow controllers where TMGa was diluted with hydrogen (H_2_) as a carrier gas. We used a pressure gauge in the microscope column next to the pole piece to monitor the total pressure and calculated the partial pressure at the sample using internal calibrations presented elsewhere.^[^ ^27^ ^]^ The growth temperature maintained at the cantilever was calibrated in two ways: ex situ by Raman spectroscopy and in situ using the AuSi eutectic melting point at 363 °C of the Au catalysts and the Si microheater sidewall. Overall, the temperature had an estimated uncertainty of ±25 °C. Detailed information on the microheater design, fabrication, and calibration is presented as part of the Experimental Section and the Supporting Information (Sections S1–S3).

The selectivity in crystal phase formation was mapped over a range of precursor flows and temperatures from 420 °C to 495 °C. The mass flow of AsH_3_ was changed from 0.3 to 1.8 sccm with partial pressure ranging from 0.13 to 1.29 Pa, while the mass flow of TMGa was kept constant at 0.3 sccm. The corresponding TMGa partial pressure is AsH_3_ dependent and estimated to be 0.6‐1.4 × 10^−3^ Pa. This corresponds to nominal [V]/[III]‐ratios of 224 to 1345 calculated from the partial pressures. Further descriptions of the used parameter ranges and estimates are given in the following sections and the Supporting Information (Sections S1–S3).

### 2.1 Crystal Phase Map

**Figure 2:**
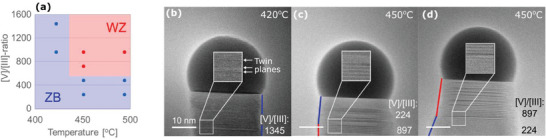
The dominating crystal phases at different group V‐flows and temperatures a). Electron micrographs illustrate the nanowires with the dominating crystal phase indicated with ZB in blue and WZ in red at different growth conditions b–d). Insets highlight regions with twin planes in ZB (b) and transitions in crystal phases from WZ to ZB (c) and ZB to WZ (d) when changing the III/V ratio as indicated.

Depending on the preset growth conditions, we observed either WZ or ZB to predominantly form by their distinct appearance in the TEM images and used this information to create a “crystal phase map”. A more detailed description of the categorization of the crystal phases is reported in the Supporting Information (Sections S5–S6). This crystal phase map is shown in **Figure** **2**a for each set of investigated growth temperature and precursor flows represented by the nominal [V]/[III]‐ratio. The map indicates the stable structure observed after a transition period as described later. ZB was formed as the exclusive crystal phase at 420 °C for the full range of AsH_3_ flows investigated, while WZ was absent under these low‐temperature conditions. Frequent twin plane formation was observed as displayed in the inset of Figure 2b. Increasing the temperature to 450 °C resulted in large amounts of stacking faults and twin planes. Further elevation of the temperature along with increased AsH_3_ supply, increasing [V]/[III]‐ratio, resulted in a crystal dominated by the WZ phase as displayed in Figure 2c,d. The results highlight that ZB is the dominating crystal phase at low temperatures and low supply of AsH_3_ (Ga‐rich environments), while WZ dominates at higher temperatures and higher AsH_3_ flows (495 °C, 897 [V]/[III]‐ratio).

A systematic map of the crystal phases formed at different temperatures and precursor flows has not been reported in the literature previously for these growth conditions and studied in situ. However, other in situ studies have reported a crystal phase shift when changing precursor flow, in agreement with our observations.^[^ ^21^, ^22^ ^]^ The characteristics of the crystal phase map with ZB forming at low temperatures and a low [V]/[III] ratio also correspond to ex situ MBE studies with a low supply of AsH_3_.^[^ ^30^, ^31^ ^]^ Conventional ex situ MOVPE systems are typically Ga‐limited, which therefore are more likely to give the contrary behavior with WZ forming at high temperatures and a low [V]/[III]‐ratio, but making an unlikely Ga‐rich growth environment for ex situ MOVPE growth the same trend as reported here has also been observed.^[^ ^18^, ^19^ ^]^ It can be explained by the fact that the total pressure of the ETEM is approximately 1 Pa, making the growth environment similar to that of an MBE system, and the supplied total pressure is significantly lower compared to the ex situ MOVPE where total pressures of 10 kPa are more typical. However, by accommodating the global differences in growth conditions, growth strategies, and, thus, potentially also conclusions from in situ with the advantage of having direct observations of single nanowires can be transferred directly to the ex situ MOVPE growth with potential for large‐scale fabrication.^[^ ^26^ ^]^

While temperature and precursor flows are directly accessible growth parameters, the changes in the droplet and nanowire geometry are often assessed as a visible guide to tell which crystal phase is forming. The contact angle, i.e., the angle between the droplet‐nanowire interface and the droplet surface, is such a geometrical parameter, which has been reported extensively.^[^ ^21^, ^22^, ^23^, ^32^ ^]^ In our study, from Figure S20 (Supporting Information), we determined a steady state characteristic contact angle of ≈107°, which roughly separates WZ forming at lower contact angles, while ZB was dominating at higher contact angles. Although conceptually in agreement with other in situ studies showing a cutoff angle between WZ and ZB, our observed value is below the ≈125° reported earlier.^[^ ^21^, ^22^ ^]^ It is also different from the non‐steady state characteristic contact angles ∼103° observed in Figures S21–S22 (Supporting Information), when changing crystal phase. Potential explanations could be differences in the growth conditions in different reactors, time to achieve a steady state, or contamination in the ETEM system as elaborated in the Supporting Information (Section S9). The underlying parameters actually determining the contact angle such as the droplet volume are not easily transferred to its projection as contact angle.^[^ ^31^ ^]^ A fuller theoretical understanding of the reliability of contact angle as a predictor of the crystal phase formation would be an interesting future study.

### 2.2 Flow Control

**Figure 3:**
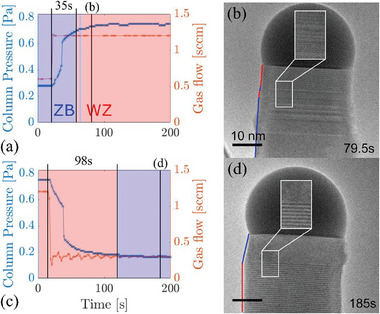
Crystal phases dominate at different flow conditions with WZ marked by red and ZB marked by blue and a constant temperature of 495 °C. The change in flow and pressure as AsH_3_ flow is increased a) and decreased c). The resulting electron micrographs of the nanowires b) and d).

Our first approach for crystal phase engineering based on the phase map is controlling the material supply flow, i.e., changing the [V]/[III]‐ratio. This has been reported to be the versatile approach ex situ. In situ, we observe a ZB to WZ transition when changing the AsH_3_ precursor flow and hence partial pressure, from low to high, while the reverse trend is induced for the opposite flow change routine. **Figure** **3** shows the time‐dependent change in AsH_3_ precursor flow and column pressure for both crystal phase switches from ZB to WZ (Figure 3a) and WZ to ZB (Figure 3c) at 495 °C. Here, the conditions/time when either WZ or ZB were formed are indicated by red (WZ) and blue (ZB) areas, respectively. HRTEM images of the nanowires after the completed precursor flow switching schemes are shown correspondingly in Figure 3b,d.

A direct correlation between the precursor gas flow and the column chamber pressure is observed in Figure 3a,c. However, the response time is ≈32–40 s after initiation of the gas flow changes to reach 90% of the corresponding column pressure value, depending on the direction of the flow change (increase or decrease) and the difference in flow. The final step of pressure stabilization from 90% to ≈100% was much faster (45 s) for an increase compared to a decrease in flow (101 s). As a result, it took ca. 35 s to start forming WZ, while more than double, 98 s, were needed for ZB to start forming. Different onset delays for the transition from ZB to WZ and vice versa have also been observed ex situ, where the change of precursor flows in the growth reactor occurs much faster than in situ, suggesting the delayed change is caused by other factors than the precursor flow.^[^ ^19^ ^]^ However, a convolution of both effects might be the case in situ: a slow response of the in situ system to flow changes combined with differences in the onset delays for WZ and ZB, respectively. To conclude from these observations: Upon changing the supply flow, the response of the system and, thus, the crystal phase formed is much slower than the time scales for the diatomic monolayer growth. This can be divided into two steps for the crystal monolayer formation in nanowires:^[^ ^25^ ^]^ 1) The incubation time is the time it takes for the droplet to form a new nucleus after the completion of a monolayer. 2) The monolayer growth time is the time it takes to complete a new monolayer after a nucleus is formed. These were observed as reported in the Supporting Information (Section S8) to be 0.8–1.6 s and 0.6–1.4 s, respectively.

Considering the purity or sharpness of the transitions, we find that shifting from ZB to WZ did result in stacking defects such as twin planes, while the slower transition from WZ to ZB rendered a sharp interface as seen in Figure 3b,d, respectively. Hence, using changes in the precursor flow for crystal phase engineering in situ, it is not only the position of the transition, which is challenging to control, but also the purity of the crystal phases forming. The abruptness of the transition can be explained by how the competing energies favoring either of the crystal phases are being balanced as discussed for temperature later.^[^ ^18^ ^]^

### 2.3 Temperature Control

To complement the crystal phase control using gas flow, temperature switching is also shown to affect the formed crystal phase.^[^ ^14^, ^15^ ^]^ While it is time‐consuming for ex situ bulk heating systems to change temperature, the microcantilever heater allows millisecond control.^[^ ^33^, ^34^ ^]^ The cantilever resistive heating is controlled by an applied bias, *V_RH_*, and a change of 100 °C can be achieved in milliseconds as calculated in the Supporting Information (Section S4). As reported from the crystal phase map in Section 2.1, such a temperature change is perfectly suitable for reaching the different crystal phases in a time shorter than the monolayer formation time.

**Figure 4:**
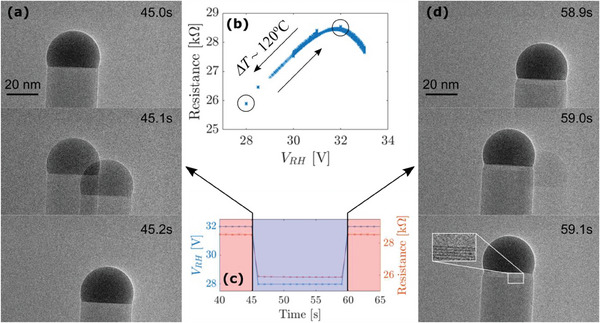
Crystal phases dominate at different temperatures. Temperatures are lowered instantaneously c) by changing bias, V_RH_, from 32 V to 28 V marked by circles in b) and lowering resistance corresponding to a temperature shift of ≈120 °C resulting in a corresponding crystal phase shift illustrated by electron micrographs a,d).

**Figure** **4** shows the experimental results of changing the temperature down and up by ≈120 °C, achieving an alternating crystal phase from ZB at the lower temperature to WZ at the higher. The temperature change causes a thermal drift within a fraction of the 0.1 s image exposure time as we observe two images of the single nanowire within one frame (middle frame of Figure 4a,d). The thermal drift makes two positions of the nanowire visible with 50% and 50% of the exposure time in the two positions in Figure 4a, and 95% and 5% in Figure 4d. There is no discernible smeared‐out image of the nanowire drifting between the two positions, and from this, we estimate the drift to happen on a time scale faster than 10% of the 0.1 s exposure or 10 ms. Within the total time scale of ≈65 s, the crystal phase goes from WZ to ZB and ZB to WZ as temperature is first decreased and then increased again. In contrast to the change in precursor flow, the time scale of the temperature change is much faster than the growth of one monolayer, and the droplet/nanowire system responds immediately forming a sharp transition from one phase to another.

**Figure 5:**
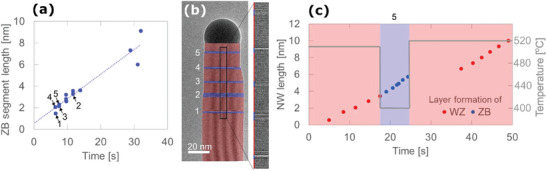
Length of ZB segments a) formed at different timespans in a nanowire with an otherwise WZ structure. A trend line indicates a small offset of 0.7 ± 0.8 nm. An electron micrograph b) of a nanowire with five segments marked 1–5 is illustrated with the different ZB segments indicated in blue. The formation of individual diatomic monolayers in the ZB segment 5 c) embedded between WZ structures counted as bilayers is plotted as a function of time showing a long incubation time as the temperature is elevated.

To achieve the desired design of the CPQDs controlling the length and the position of the QDs, both temperature and precursor flows can be used to switch the crystal phase, but it is important to choose the technically suitable parameter for which the growth system responds fast. Here the temperature offers far better control and was very successfully applied as illustrated in **Figure** **5** (Figure S23, Supporting Information), where CPQDs of different lengths have been introduced in a nanowire by temperature changes with growth times ranging from 5 to 35 s. The QD length is defined by the monolayers formed during the temperature switch and in Figure 5a, we observe a linear relation between the segment lengths and the time allowed for the QD to form. Small deviations from the linear trend could stem from the fact that we have a 30 °C uncertainty in the temperature setting for each crystal phase formed along with an uncertainty of the growth rate determination of approximately ±0.3 nm s^−1^. The temperature control and the purity of the individual segments are detailed as part of the Supporting Information (Section S8).

For perfection and application of the crystal phase quantum dots, the morphology of the nanowire as well as the purity of the crystal phases are important to consider. The nanowire diameter varies significantly along its length as visible in Figure 5b and Figure S23a–c. This variation can be primarily attributed to the following two mechanisms: An initial shrinking of the nanowire diameter due to a combined shrinking of droplet volume and an increase in contact angle (Figure S24, Supporting Information) during ZB growth, similar to observations in the literature,^[^ ^22^ ^]^ and an increase of the diameter upon radial growth of the ZB and WZ crystal phases, respectively (Figure S24 and Movie S1, Supporting Information). Higher free surface energies of ZB side facets result in faster growth rates compared to WZ, causing ZB segments to widen radially over time (Figure S24, Supporting Information). Conversely, WZ exhibits slower radial growth. However, nucleation at the ZB‐WZ interface and subsequent step flow enable the nanowire to maintain a consistent diameter across both segments, despite differences in nucleation barriers.^[^ ^18^, ^35^ ^]^ Overall, higher temperatures result in higher vapor–solid growth rates and thus, wider nanowires as illustrated by the temperature increase in Figure S23a–c. To control such radial growth, further finetuning of the low‐temperature growth should be made, when growing WZ, together with finetuning the precursor flow to minimize the droplet shrinkage and radial growth of the ZB segment.

In literature, the temperature has also been found to affect the purity of the crystal phases, where more pure phases of ZB were found at lower temperatures, while WZ was at higher temperatures.^[^ ^36^ ^]^ When changing temperatures from regions favoring WZ to ZB or vice versa, mixed phases are most likely to appear due to similar nucleation probabilities for ZB and WZ nuclei.^[^ ^17^ ^]^ The crystal phase formation is determined by the lowest nucleation barrier available for either WZ or ZB.^[^ ^37^ ^]^ This barrier is typically described by their Gibbs free energies depending on both geometrical constants, the supersaturation being the difference in chemical potential between the solid and the liquid, the difference in cohesive energy between the WZ and ZB phase (the binding energy for the atoms in the crystal lattice being higher for WZ than for ZB) and the average surface energy of the nuclei determined by the surface energies, the contact angle and the fraction of the nucleus in contact with the vapor.^[^ ^37^ ^]^ Temperature affects supersaturation, which decreases when increasing temperature at fixed concentrations.^[^ ^38^ ^]^ However, the Ga concentration also increases with higher temperature resulting in a greater volume and by this increasing the contact angle.^[^ ^24^, ^39^ ^]^ Finally, temperature also influences the growth rate similarly determining what crystal phase is most likely to nucleate. At high temperatures, the nucleation of the WZ crystal phase is likely to happen, because the low surface energy of the WZ side facets dominates due to a greater droplet volume. While ZB is favored when supersaturation dominates at the lower temperatures. Hence, temperature is a highly complex growth parameter, which from theory and observations in literature is expected to affect multiple aspects of the growth such as longitudinal and radial growth rates and the crystal phase selectivity.

In this study, the effect of temperature can be observed by studying the layer formation of WZ and ZB for each individual segment. When lowering the temperature and, thus, going from WZ to ZB, the initial step finishes with the started crystal phase, WZ in this case, even though the temperature is changed. For the opposite direction, namely increasing the temperature and going from ZB to WZ, the growth generally stops, and a longer incubation time is observed for the initial WZ layer to form as illustrated in Figure 5c. The growth stop could be explained by the supersaturation of the droplet with respect to the solid being temporarily lowered by the increase in temperature.^[^ ^24^, ^38^ ^]^ As the supersaturation is being rebuilt the volume increases as the influx of material to the liquid is relatively unchanged, while the growth is stopped (Figure S22, Supporting Information). In some cases, not only growth interrupts are observed but even etching sometimes occurs. For two of the longer segments, 3–4 monolayers of ZB are removed, before growth of WZ is initiated again, which is an important finding, considering the ultimate control aimed at. In general, for the engineering of specific‐sized CPQDs, this delay offset and possible etching when increasing temperature should be monitored to achieve the ultimate control of the quantum dots. Another important observation for determining the CPQD size and position is the growth rate. This is known to increase with increasing supersaturation and temperature.^[^ ^37^ ^]^ From Figure 5c, it may seem like ZB has a higher growth rate than WZ, but we also observe the contrary, which is elaborated further in the Supporting Information (Figure S25, Supporting Information). At high temperatures, the growth rates increase for both the WZ and ZB. Figure S25c shows that **the** growth rate is not significantly different for WZ and ZB at the temperatures in this study.

The observations of growth interruptions and/or continuous growth upon substantial temperature changes can give some valuable insight into the growth mechanism. As mentioned, we observe a delay in growth when transitioning from ZB to WZ as growth interrupts due to increasing temperature (Figure 5c). With an associated increase in the equilibrium concentration of Ga, this delay is likely to occur as a result of the supersaturation momentarily falling below the critical level for nucleation. On the other hand, when the temperature is lowered, the equilibrium concentration of Ga decreases. Thus, supersaturation momentarily increases with respect to the critical concentrations for nucleation, and the crystal is therefore continuously grown resulting in a small positive offset (Figure 5a). As such, we observe continuous growth as the conditions change from favoring WZ to ZB. However, the offset behavior is related to the inherent aspect of crystal growth being driven by supersaturation but not necessarily to the crystal phases formed. A similar behavior has been observed for the reversed process, the decomposition of GaAs, where the decomposition rate alters momentarily as a result of changes in supersaturation.^[^ ^40^ ^]^ The delay has been previously reported for ex situ experiments when a change from ZB to WZ has been induced by decreasing the AsH_3_ flow at a constant growth temperature.^[^ ^19^ ^]^ A reduced AsH_3_ flow can cause the Ga concentration within the droplet to increase to reach a new equilibrium concentration as reported by Maliakkal et al.^[^ ^24^ ^]^ and, thus, momentarily decrease the supersaturation, explaining the observed offset in the crystal growth initiation. The difference in the Ga concentration and the equilibrium concentration may even lead to the dissolution of a few monolayers of the GaAs nanowire, which can explain the observations for some of the segments with higher temperature differences (Figure 5a). These results bridge an important gap of understanding the role between supersaturation and temperature to existing observation of precursor effect, and to new ways to unravel the dynamics of supersaturation.

To conclude, the current study using a microfabricated cantilever as a µMOVPE system can help with a fundamental understanding of in situ growth. It further provides significant advantages for crystal phase engineering with atomic precision and emphasizes the importance of in situ TEM observations where, however, the irradiation may influence the observed processes. In our case, e.g., the CPQD nanowire shown in Figure 5 had a neighboring nanowire (<1 µm away) that was less affected by irradiation and had somewhat similar CPQD segments but not identical. Hence, the beam influence should be more closely studied in the future. It also remains to be studied how in situ grown CPQD performs optically and if coatings/annealing are needed, or ex situ µMOVPE is needed for optimally functional optical devices. The temperature control can potentially be transferred to ex situ growth systems to overcome bulk heating and ensure local growth for, e.g., device fabrication.^[^ ^28^ ^]^ Finally, it allows for electrical contacts to the nanowires,^[^ ^41^, ^42^ ^]^ is complementary metal–oxide–semiconductor (CMOS) compatible,^[^ ^28^ ^]^ and can in principle hold nanowires both with back‐gates on an insulating membrane or freely suspended nanowire bridges, which may be the perfected noise free CPQD system, which could be relevant for device fabrication. The system could also be extended and used for other nanoscale temperature‐dependent processes.

## 3 Conclusion

In summary, using microcantilever heaters for epitaxial III‐V growth in a combined ETEM‐MOVPE system, we have mapped the crystal phase formation at different precursor flows and temperatures for GaAs nanowire crystal phase engineering to achieve atomic‐scale control of the position and length of CPQDs. Zincblende is generally formed at low temperatures and low arsine flows, while wurtzite is the dominating crystal phase at high temperatures and high arsine flows in the overall Ga‐rich environment of the in situ growth environment. As discussed, with due consideration of differences between in situ and ex situ growth conditions, growth strategies and, thus, potentially also conclusions from direct observations in situ can be transferred directly to the ex situ MOVPE growth with the potential to aid in optimizing large‐scale fabrication. The micrometer size of the cantilevers allows temperature shifts of more than 100 °C within 1 ms at the nanowire growth temperature, which can be much faster than the diatomic monolayer growth rate. This is majorly different from the precursor flow changes taking tens of seconds to respond. The formation of µMOVPE CPQDs by temperature is found to be superior in control and purity compared to the precursor flow control in situ. Both approaches to engineer the crystal phase, changing temperature or precursor flow could be explained by the resulting effect on the supersaturation of the catalyst droplet. These findings enable the engineering of multiple CPQDs with atomically precise positions to potentially investigate their interactions. Apart from the findings on CPQD formation in GaAs NWs, the approach could easily be extended to composition and catalyst characterization in this and other nanostructured systems. Even further, one could think of investigating time‐ and temperature of, e.g., phase transition at the nanoscale.

## 4 Experimental Section

### Microheater Fabrication

The microheater device was fabricated on a specially manufactured (110) silicon‐on‐insulator (SOI) wafer. This consisted of a few micrometers, 3.5‐4.5 µm, thin device layer being p‐doped Si to lower resistivity, *ρ* = 0.05−0.15 Ω‐cm, resulting in a high conductivity, σ = 1/ρ. The <111> crystal direction was perpendicular to the sidewall between two neighboring microheaters, when the device was fabricated with a specific alignment. Below the conducting device layer was a thin insulating layer (475−525 nm) of buried oxide, SiO_2_, and the rest of the chip base consisted of a 290–310 µm thick handle layer of Si with a high resistivity of 520 Ω‐cm. The microfabrication of the devices was based on previous process procedures.^[^ ^29^ ^]^ It was achieved by UV‐lithography‐defined photoresist as etch mask for a high‐aspect‐ratio SF_6_/C_4_F_8_‐based reactive ion etching (RIE) process. The center hole of the chips with the microheaters was formed by a 150‐nm stoichiometric low‐pressure (LP) CVD silicon nitride layer deposited on both sides of the wafer. The backside nitride was patterned lithographically and used as an etch mask for an anisotropic potassium hydroxide etch of the handle wafer with the buried oxide as an etch stop. A low resistance electrical contact to the microheaters was obtained by metal deposition of 5 nm Ti and 50 nm Au at the contact pads and electrical leads using UV‐lithography photoresist as mask on the device and a lift‐off procedure after metal deposition. The wafer was cut to chips fitting the TEM sample holder of the ETEM.^[^ ^43^ ^]^ A full description of the microheater design and process flow can be found in the Supporting Information (Sections S1 and S2). The electrode contact pads on the MEMS could be connected to four pins from the sample holder, which could be controlled by two Keithley SourceMeters (type 2611B and 2400). The applied biases and resulting current were managed by LabVIEW software developed for each type of Keithley.

### TEM Specifications

The ETEM used was a Hitachi HF3300S TEM with an acceleration voltage of up to 300 kV.^[^ ^27^, ^43^ ^]^ The electron source was a cold FEG, which demanded ultra‐high vacuum conditions to keep the filament clean to ensure high control of electron extraction. Higher pressure was possible at the sample region because of three ion pumps installed in the microscope column lowering the pressure closer to the gun region. A CEOS BCOR aberration corrector was installed to improve the resolution to an information limit of 86 pm, but the true resolution after correction was more likely 1 Å with almost no temperature and pressure dependency for temperatures below 600 °C and pressures below 1 Pa. Images and videos were captured with a Gatan OneView IS camera with a CMOS sensor up to size 4096 by 4096 pixels and a pixel size of 15 µm. Samples were loaded into the microscope using a sample holder with two tilting directions, α and β. An integrated EDX‐detector (Oxford Instruments X‐Max) was used to measure the droplet and nanowire compositions. More details can be found elsewhere.^[^ ^27^, ^43^ ^]^

### Precursor Supply

The microscope allowed Metal‐Organic Vapor Phase Epitaxy (MOVPE) at the sample region by a purpose‐built gas handling system led by two separate side port injections. The gas system had access to the metalorganic group III and group V precursors: trimethylgallium (TMGa), trimethylindium (TMIn), trimethylaluminium (TMAl) and trimethylstibine (TMSb) and the hydride group V precursors: arsine (AsH_3_) and phosphine (PH_3_) as well as the standard gases: Hydrogen (H_2_), nitrogen (N_2_) and oxygen (O_2_). The hydride precursors (AsH_3_ and PH_3_) were guided directly to the sample area in their vapor phases, while the metalorganic groups were added from a bubbler at a controlled temperature using H_2_ carrier gas. The precursors were further diluted by H_2_ before reaching the sample. Part of the calculated partial pressure variation described in Section 2 comes from how the pressure gauge on the column works. Since it is a gas‐type dependent gauge with dual measurement techniques, it is not linear in the H_2_/AsH_3_ gas mixture in this pressure range. Hence, the TMGa partial pressure was not able to more accurately state for different AsH_3_ flows. The nominal V/III ratio is of the partial pressures, which makes the results comparable across growth methods and it is often different from the mass flow ratio. More details regarding the microscope and the calibrations of the partial pressure at the sample position can be found elsewhere.^[^ ^27^ ^]^

### Temperature Calibration

The temperature of the microheaters was controlled by resistive heating applying a bias to the microheater loop. The tip temperature can be estimated using the resistance peak at thermally excited carrier runaway as a calibration point when applying a bias to the microheater.^[^ ^28^ ^]^ The tip temperature at this calibration point has been estimated to be 475–525 °C, while linear coefficients were determined to be 27–31 °C V^−1^ for the linear region before and 30–78 °C V^−1^ after the resistance peak. This has been determined by ex situ calibration methods comparing Raman spectroscopy in air, FEM simulations in air and vacuum, and finally, a linear extrapolation of the AuSi‐melting point found in situ. Another in situ temperature calibration method was investigated using the AuGa catalyst of the nanowires, which only depends on the atmospheric conditions and the lack of contamination in the liquid droplet. This was an attractive method once nanowires were growing. The temperature calibration methods and results using these are summarized in the Supporting Information (Section S3).

### Data Processing

For Figure 1, 2, 3, 4, 5 and Figure S1–S29 (Supporting Information), *DigitalMicrograph* from *Gatan* was used to analyze High Resolution TEM (HRTEM) images and extract HRTEM images from the HRTEM movies. The crystal phase categorization was done manually as described in the Supporting Information (Section S5). The data visualization was done using *MATLAB R2022b*. The compositional analysis from the EDX measurements was done using *Aztec* software. The figures in the main text and the Supporting Information were prepared using *InkScape*.

## Conflict of Interest

The authors declare no conflict of interest.

## Supporting information



Supporting Information

Supplemental Movie 1

## Data Availability

The data that support the findings of this study are available from the corresponding author upon reasonable request.

## References

[1] H. J. Joyce , Q. Gao , H. Hoe Tan , C. Jagadish , Y. Kim , J. Zou , L. M. Smith , H. E. Jackson , J. M. Yarrison‐Rice , P. Parkinson , M. B. Johnston , Prog. Quantum Electron. 2011, 35, 23.

[2] M. Scandi , D. Barker , S. Lehmann , K. A. Dick , V. F. Maisi , M. Perarnau‐Llobet , Phys. Rev. Lett. 2022, 129, 270601. 36638287 10.1103/PhysRevLett.129.270601

[3] M. S. Lozano , V. J. Gómez , Nanoscale Adv 2023, 5, 1890. 36998660 10.1039/d2na00956k PMC10044505

[4] D. Spirkoska , J. Arbiol , A. Gustafsson , S. Conesa‐Boj , F. Glas , I. Zardo , M. Heigoldt , M. H. Gass , A. L. Bleloch , S. Estrade , M. Kaniber , J. Rossler , F. Peiro , J. R. Morante , G. Abstreiter , L. Samuelson , A. Fontcuberta I Morral , Phys. Rev. B 2009, 80, 245325.

[5] N. Akopian , G. Patriarche , L. Liu , J. C. Harmand , V. Zwiller , Nano Lett. 2010, 10, 1198. 20205446 10.1021/nl903534n

[6] N. Vainorius , S. Lehmann , D. Jacobsson , L. Samuelson , K. A. Dick , M. E. Pistol , Nano Lett. 2015, 15, 2652. 25761051 10.1021/acs.nanolett.5b00253

[7] B. Loitsch , J. Winnerl , G. Grimaldi , J. Wierzbowski , D. Rudolph , S. Morkötter , M. Döblinger , G. Abstreiter , G. Koblmüller , J. J. Finley , Nano Lett. 2015, 15, 7544. 26455732 10.1021/acs.nanolett.5b03273

[8] I. Geijselaers , N. Vainorius , S. Lehmann , C. E. Pryor , K. A. Dick , M. E. Pistol , Appl. Phys. Lett. 2021, 119, 263102.

[9] J. Hastrup , L. Leandro , N. Akopian , Sci. Rep. 2020, 10, 14911. 32913255 10.1038/s41598-020-71601-x PMC7483522

[10] G. Priante , G. Patriarche , F. Oehler , F. Glas , J. C. Harmand , Nano Lett. 2015, 15, 6036. 26217912 10.1021/acs.nanolett.5b02224

[11] T. B. Hoang , A. F. Moses , L. Ahtapodov , H. Zhou , D. L. Dheeraj , A. T. J. Van Helvoort , B.‐O. Fimland , H. Weman , Nano Lett. 2010, 10, 2927. 20604543 10.1021/nl101087e

[12] E. Luna , Á. Guzmán , A. Trampert , G. Álvarez , Phys. Rev. Lett. 2012, 109, 126101. 23005962 10.1103/PhysRevLett.109.126101

[13] C. Y. Yeh , Z. W. Lu , S. Froyen , A. Zunger , Phys. Rev. B 1992, 46, 10086. 10.1103/physrevb.46.10086 10002848

[14] P. Caroff , K. A. Dick , J. Johansson , M. E. Messing , K. Deppert , L. Samuelson , Nature Nanotech 2009, 4, 50. 10.1038/nnano.2008.359 19119283

[15] H. J. Joyce , J. Wong‐Leung , Q. Gao , H. Hoe Tan , C. Jagadish , Nano Lett 2010, 10, 908. 20131909 10.1021/nl903688v

[16] K. A. Dick , J. Bolinsson , M. E. Messing , S. Lehmann , J. Johansson , P. Caroff , J. Vac. Sci. Technol. B 2011, 29, 04D103.

[17] S. Assali , L. Gagliano , D. S. Oliveira , M. A. Verheijen , S. R. Plissard , L. F. Feiner , E. P. A. M. Bakkers , Nano Lett. 2015, 15, 8062. 26539748 10.1021/acs.nanolett.5b03484

[18] S. Lehmann , J. Wallentin , D. Jacobsson , K. Deppert , K. A. Dick , Nano Lett. 2013, 13, 4099. 23902379 10.1021/nl401554w

[19] S. Lehmann , D. Jacobsson , K. A. Dick , Nanotechnology 2015, 26, 301001. 26160888 10.1088/0957-4484/26/30/301001

[20] D. Anandan , V. Nagarajan , R. K. Kakkerla , H. W. Yu , H. L. Ko , S. K. Singh , C. T. Lee , E. Y. Chang , J. Cryst. Growth 2019, 522, 30.

[21] D. Jacobsson , F. Panciera , J. Tersoff , M. C. Reuter , S. Lehmann , S. Hofmann , K. A. Dick , F. M. Ross , Nature 2016, 531, 317. 26983538 10.1038/nature17148 PMC4876924

[22] F. Panciera , Z. Baraissov , G. Patriarche , V. G. Dubrovskii , F. Glas , L. Travers , U. Mirsaidov , J.‐C. Harmand , Nano Lett. 2020, 20, 1669. 32027145 10.1021/acs.nanolett.9b04808

[23] J.‐C. Harmand , G. Patriarche , F. Glas , F. Panciera , I. Florea , J.‐L. Maurice , L. Travers , Y. Ollivier , Phys. Rev. Lett. 2018, 121, 166101. 30387660 10.1103/PhysRevLett.121.166101

[24] C. B. Maliakkal , D. Jacobsson , M. Tornberg , A. R. Persson , J. Johansson , R. Wallenberg , K. A. Dick , Nat. Commun. 2019, 10, 4577. 31594930 10.1038/s41467-019-12437-6 PMC6783420

[25] C. B. Maliakkal , E. K. Mårtensson , M. U. Tornberg , D. Jacobsson , A. R. Persson , J. Johansson , L. R. Wallenberg , K. A. Dick , ACS Nano 2020, 14, 3868. 32049491 10.1021/acsnano.9b09816 PMC7307954

[26] M. Marnauza , M. Tornberg , E. K. Mårtensson , D. Jacobsson , K. A. Dick , Nanoscale Horiz. 2023, 8, 291. 36621012 10.1039/d2nh00432a

[27] M. Tornberg , C. B. Maliakkal , D. Jacobsson , R. Wallenberg , K. A. Dick , Microsc. Microanal. 2022, 28, 1484. 10.1017/S1431927622000769 35644630

[28] K. Mølhave , B. A. Wacaser , D. H. Petersen , J. B. Wagner , L. Samuelson , P. Bøggild , Small 2008, 4, 1741. 18819133 10.1002/smll.200800366

[29] C. Kallesøe , C. Y. Wen , K. Mølhave , P. Bøggild , F. M. Ross , Small 2010, 6, 2058. 20730823 10.1002/smll.200902187

[30] M. C. Plante , R. R. LaPierre , Nanotechnology 2008, 19, 495603. 21730678 10.1088/0957-4484/19/49/495603

[31] F. Glas , J. C. Harmand , G. Patriarche , Phys. Rev. Lett. 2007, 99, 146101. 17930689 10.1103/PhysRevLett.99.146101

[32] W. Kim , V. G. Dubrovskii , J. Vukajlovic‐Plestina , G. Tütüncüoglu , L. Francaviglia , L. Güniat , H. Potts , M. Friedl , J. B. Leran , A. Fontcuberta I Morral , Nano Lett. 2018, 18, 49. 29257895 10.1021/acs.nanolett.7b03126

[33] J. Y. Howe , M. S. Thompson , S. Dogel , K. Ueda , T. Matsumoto , H. Kikuchi , M. Reynolds , H. Hosseinkhannazer , T. J. Zega , Microsc. Microanal. 2017, 23, 66.

[34] J. T. van Omme , M. Zakhozheva , R. G. Spruit , M. Sholkina , H. H. Pérez Garza , Ultramicroscopy 2018, 192, 14. 29802911 10.1016/j.ultramic.2018.05.005

[35] S. Lehmann , D. Jacobsson , K. Deppert , K. A. Dick , Nano Res. 2012, 5, 470.

[36] S. Paiman , Q. Gao , H. J. Joyce , Y. Kim , H. H. Tan , C. Jagadish , X. Zhang , Y. Guo , J. Zou , J Phys D Appl Phys 2010, 43, 445402.

[37] E. K. Mårtensson , S. Lehmann , K. A. Dick , J. Johansson , Nano Lett. 2019, 19, 1197. 30618259 10.1021/acs.nanolett.8b04637

[38] F. Glas , J. Appl. Phys. 2010, 108, 073506.

[39] T. Dursap , M. Vettori , A. Danescu , C. Botella , P. Regreny , G. Patriarche , M. Gendry , J. Penuelas , Nanoscale Adv 2020, 2, 2127. 36132505 10.1039/d0na00273a PMC9418245

[40] M. Tornberg , D. Jacobsson , A. R. Persson , R. Wallenberg , K. A. Dick , S. Kodambaka , Nano Lett. 2019, 19, 3498. 31039317 10.1021/acs.nanolett.9b00321

[41] S. B. Alam , F. Panciera , O. Hansen , K. Mølhave , F. M. Ross , Nano Lett. 2015, 15, 6535. 26367351 10.1021/acs.nanolett.5b02178

[42] S. B. Alam , C. R. Andersen , F. Panciera , A. A. S. Nilausen , O. Hansen , F. M. Ross , K. Mølhave , Nanotechnology 2020, 31, 494002. 32746444 10.1088/1361-6528/ababc8

[43] C. J. D. Hetherington , D. Jacobsson , K. A. Dick , R. Wallenberg , Semicond. Sci. Technol. 2020, 35, 034004.

