# Supplementary material for: Microheater Controlled Crystal Phase Engineering of Nanowires Using In Situ Transmission Electron Microscopy
Source: Small Methods. 2024 Sep 23;9(1):2400728. doi: 10.1002/smtd.202400728 (PMC11740929; doi:10.1002/smtd.202400728)
Supplement: Supplementary file 1 — Supporting Information [file SMTD-9-2400728-s002.docx]

(SI) Microheater controlled crystal phase engineering of nanowires using in situ Transmission Electron Microscopy

Christopher R.Y. Andersen, Marcus Tornberg, Sebastian Lehmann, Daniel Jacobsson, Kimberly A. Dick*, Kristian S. Mølhave*

Contents

[1. Microheater design 2](#_Toc171627647)

[2. Process flow 2](#_Toc171627648)

[3. Temperature calibration methods 5](#_Toc171627649)

[3.1 By Raman Spectroscopy 5](#_Toc171627650)

[3.2 By Finite Element Modeling 6](#_Toc171627651)

[3.3 By Catalyst Melting Point 8](#_Toc171627652)

[3.4 By Droplet Composition 10](#_Toc171627653)

[3.5 Summary on temperature calibration methods 11](#_Toc171627654)

[4. Calculations of temperature change 13](#_Toc171627655)

[5. Crystal phase categorization 14](#_Toc171627656)

[6. Crystal phase map 15](#_Toc171627657)

[7. Droplet Volume and Contact Angles 15](#_Toc171627658)

[8. Crystal Phase Quantum Dots 17](#_Toc171627659)

[9. Droplet and nanowire composition 19](#_Toc171627660)

[10. References 20](#_Toc171627661)

# Microheater design


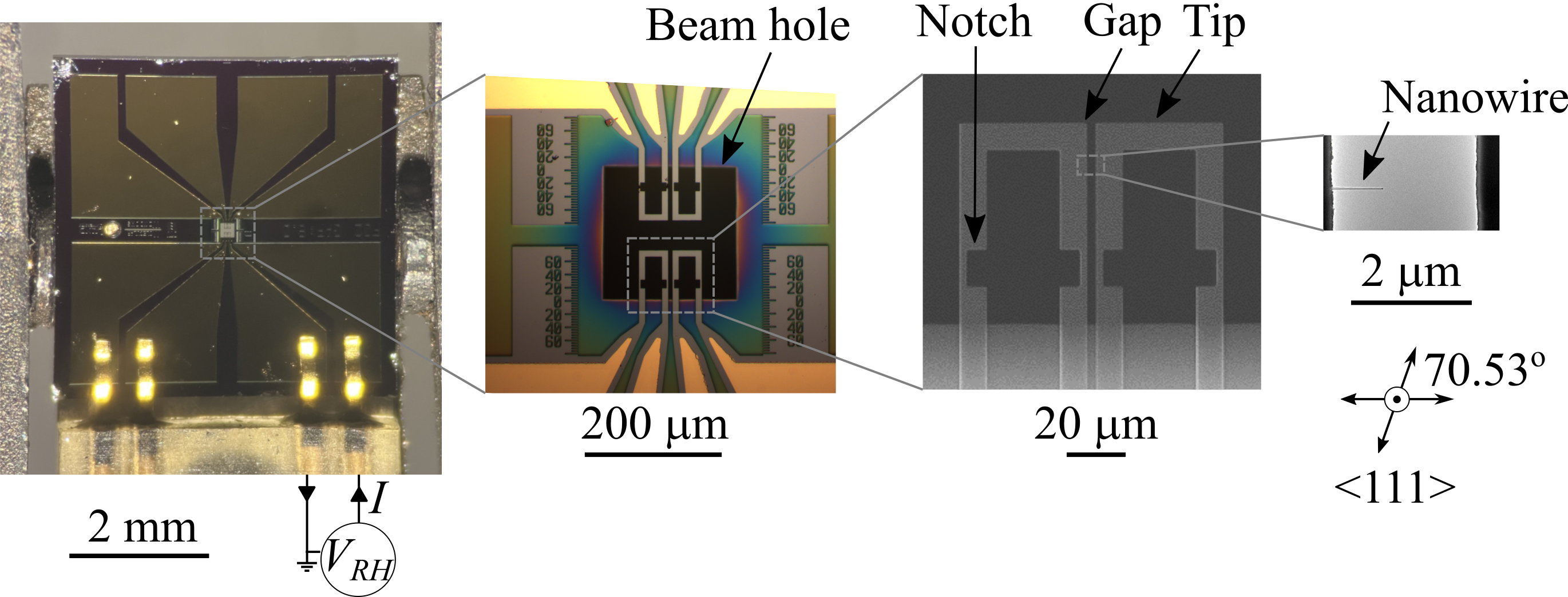


**Figure S1.** Overview of chip in a TEM sample holder with four electrical contact bins. The beam hole is illustrated with four microheaters together with the microheater design. The <111> crystal directions on the microheater sidewalls are indicated with a low magnification image of a nanowire.

The microheater design is illustrated in **Figure S1**. The chip has a size of 5 x 5 mm^2^ with four electrical contact pads, which can be accessed by the ETEM sample holder. The electrical contact pads lead up to two neighboring microheater cantilever loops hanging into vacuum from the chip base at the chip center working as beam hole for the electron beam. The microheater loop has one notch at each side with a narrower region of the silicon. This results in a higher thermal resistance than the rest of the microheater loop leading to a fairly uniform and high temperature increase in the region between the notches. The distance from the notch to the chip base is considered as the offset. The greater the offset, the less of the chip base will be affected by the elevated temperature; however, a large offset also decreases the mechanical stability of the microheaters hanging into free space. An offset of 20 μm was found to be reasonable to balance these considerations. Every chip has two neighboring microheaters at each side so that a chip can be rotated 180^o^* *giving access to four microheaters in total for a sample holder with four electrical contact pins. The distance between two neighboring microheaters is noted as the gap, which was most likely 2.0-2.5 μm set by UV lithography limitations.

# Process flow

The process flow consisted of three parts: Fabrication of (1) the Device, (2) Handle and (3) Metal Contact layer using a specially manufactured (110) silicon-on-insulator (SOI) wafer (See **Figure S2** to get an overview of the layers). The device layer consisted of a few micrometers, 3.5-4.5 μm, thin device layer being p-doped Si to lower resistivity, *ρ* = 0.05-0.15 Ω-cm, resulting in a high conductivity, σ = 1/ρ. The device was fabricated with a specific alignment to ensure nanowire growth bridging two cantilevers, where the <111> crystal direction was perpendicular to the sidewall between two neighboring microheaters. The procedure for fabricating the microheaters and structures for the electrical contact (as shown in **Figure S1**) is illustrated and described in **Figure S2.**

The handle layer consisting of 290-310 μm Si with a high resistivity of 520 Ω-cm was processed to form membranes from the insulating layer. The procedure is illustrated and described in **Figure S3**.

The electrical contact to the MEMS chips was improved by adding a metal layer of Ti/Au on top of the device layer. This is illustrated and described in **Figure S4**.

Before use, the protective resist is removed in solvents. Then catalytic Au particles are deposited from an aerosol source to cover the surface. The chip is then placed on a special screw cap with a hole drilled in it on an HF solution bottle, while observing in a microscope. This allows a vapor HF etch of the suspended SiO_2_ layer from below the chip, leading to suspended cantilevers and the SiO_2_ remaining on the major part of the chip to ensure functionality. The chip is quickly inserted into the TEM after HF etch to avoid formation of native oxide.


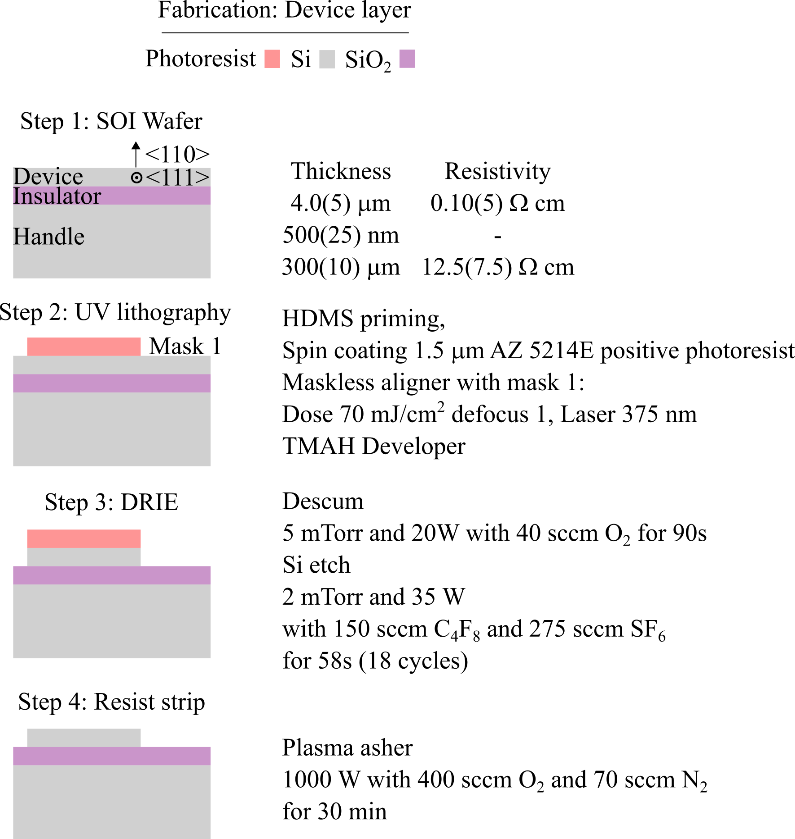


**Figure S2.** The fabrication of the device layer.


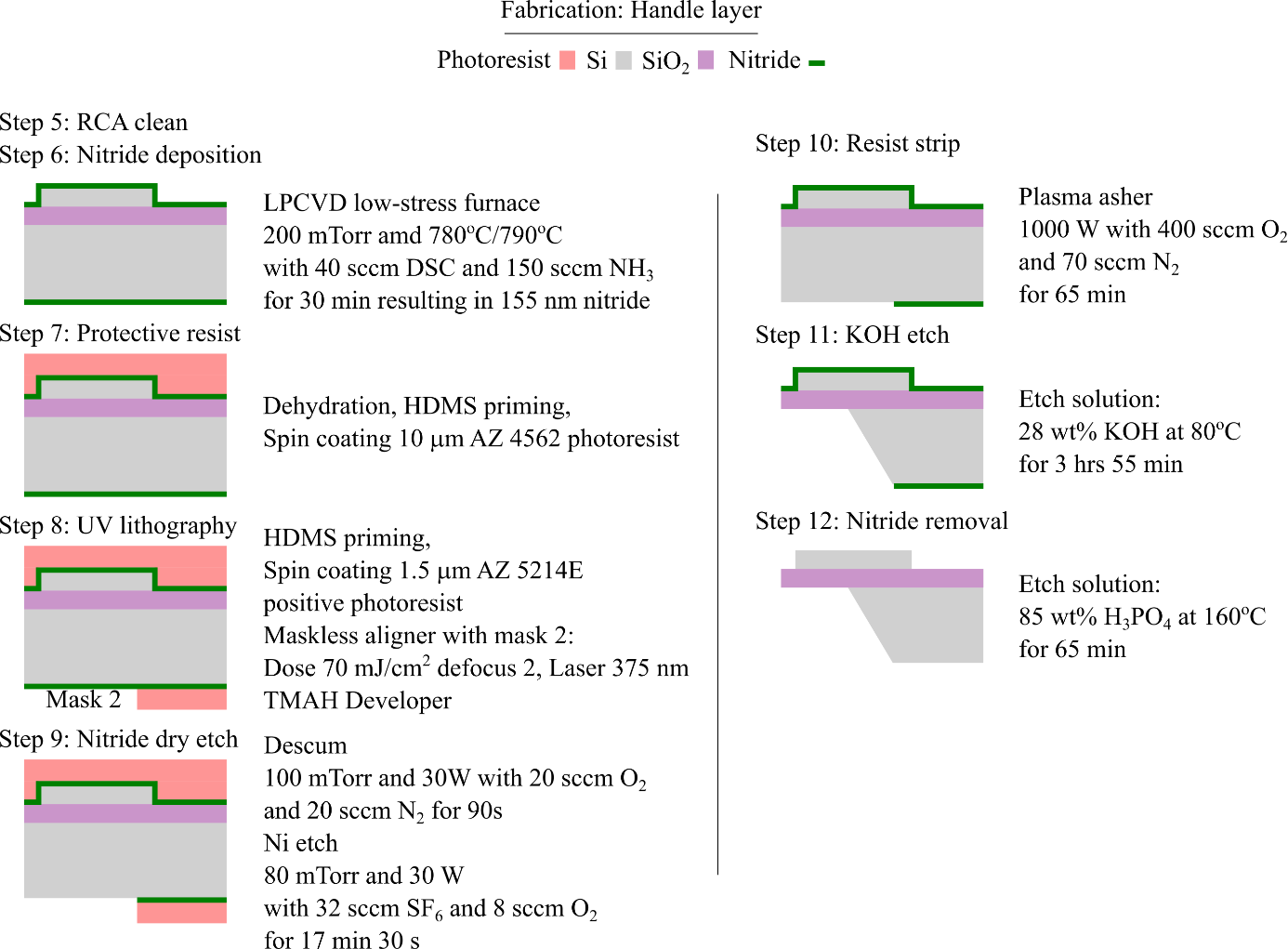


**Figure S3.** The fabrication of the handle layer.


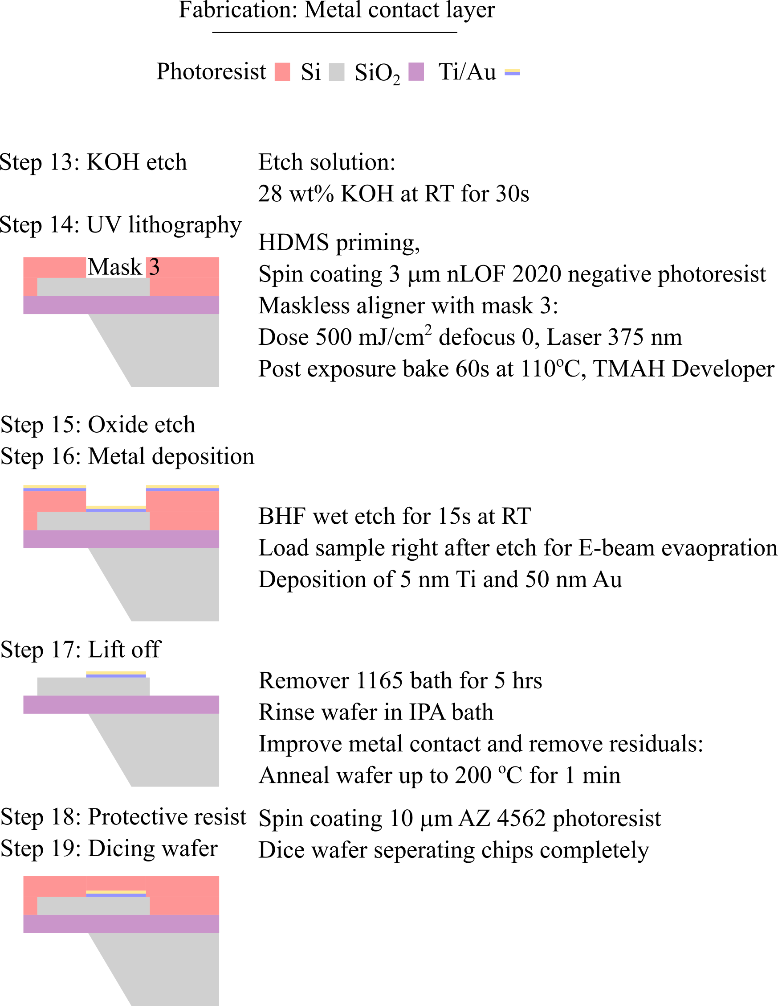


**Figure S4.** The fabrication of the metal contact layer.

# Temperature calibration methods

We use three calibration methods to estimate temperature: Raman spectroscopy calibration ex situ (Section 3.1), Finite Element Model simulations with vacuum and air environments corresponding to in situ and ex situ conditions (Section 3.2) and the melting point of the eutectic in situ (Section 3.3). For completeness of possible calibration methods, we also describe a fourth method using the droplet composition (Section 3.4) and suggest other calibration methods (Section 3.5). The calibration methods are compared for the calibration of the temperatures for the experiments carried out in this study (Section 3.5).

## 3.1 By Raman Spectroscopy

A common calibration technique is Raman spectroscopy, which has been used to determine temperature distribution with micrometer spatial resolution in diamond structures^[1]^, Si-^[2–4]^ and III–V semiconductors^[5–8]^. The Raman spectrometer used in this Paper could not measure the anti-Stokes signal and was operated in air.

The temperature was calibrated by measuring the difference in Raman shift shown in Figure S5, ΔΩ, of the Stokes peak position at elevated temperatures, Ω (*T_high_*), compared to the peak at room temperature, Ω (*T_0_*). This approach has previously been used for undoped crystalline Si peaks from room temperature and up to 1000^o^C.^[9,10]^ The peak shift of the Stokes signal was calibrated using a Raman spectrometer with a laser wavelength of 455 nm and a power of 2.5 mW like previous calibration experiments.^[11,12]^ The resulting spot size was 0.5 μm and the accumulation time was 30 s. The Si peak was measured at the chip base at elevated temperatures up to 520^o^C using a Linkam stage to control T and vacuum. As shown in **Figure S5a**. A linear response approximation of the peak positions is considered as a reasonable approach for calibrating the peak shift as a function of temperature in an eternally heated chip with known temperature given the data in **Figure S5b** with a linear decrease with a linear coefficient of -0.0223 ± 5 cm^-1^ K. This seems to be a reasonable value compared to the peak shift calibration with literature and theory.

With resistive heating bias V_RH_ on a cantilever in vacuum we find a change in tip temperature as shown in **Figure S5c** that can be compared to other temperature assessment methods as described below.


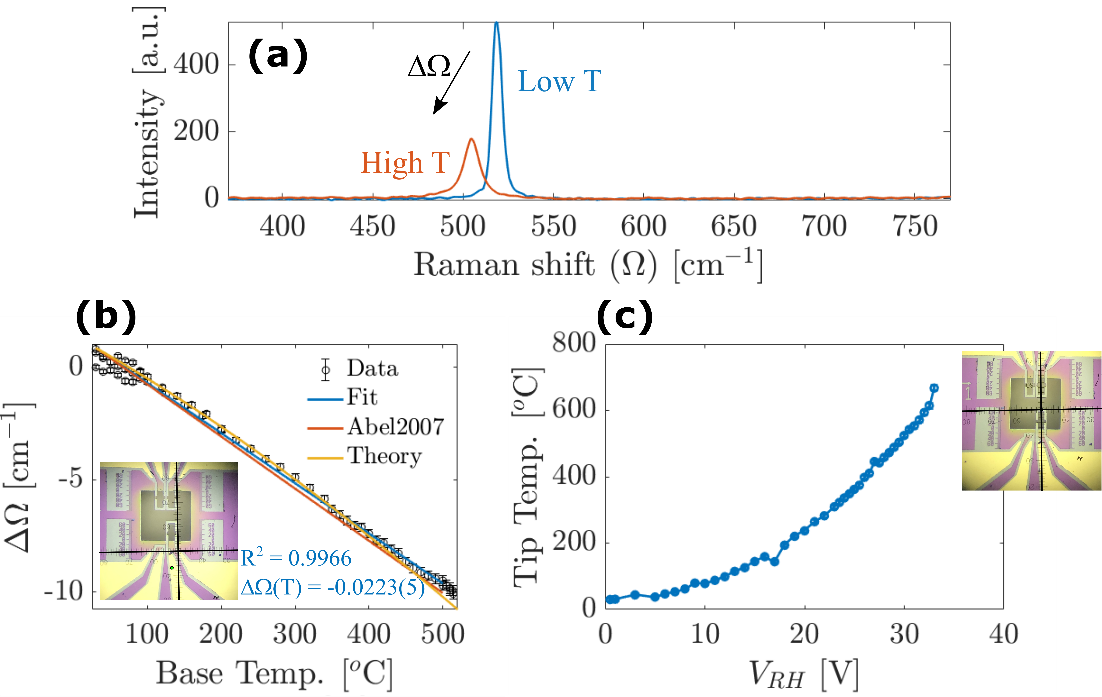


**Figure S5.** Raman spectroscopy showing the Stokes peak of the Si-microheater tip at room temperature and elevated temperature (a), a linear fit of the peak shift as function of temperature with the laser-illuminated region illustrated in the inset (b) and the tip temperature found from the peak shift of the microheater tip and the linear coefficient for the temperature (c).

## 3.2 By Finite Element Modeling

The microheater system was simulated using COMSOL Multiphysics® Version 5.6^[13]^ with the modules: Solid Mechanics, Heat Transfer in Solids and Electric Currents. These were coupled using the Thermal Expansion and the Electromagnetic Heating modules. A simple chip geometry was modelled using the dimensions of the microheater and Au-leads for a chip measured by SEM imaging and modelled in COMSOL (**Figure S6** and **Figure S7**). The leads were extended 500 μm to include their contribution to resistive heating. The insulating layer and the handle layer were added below the device and the hole from KOH-etching for the microheaters were included.


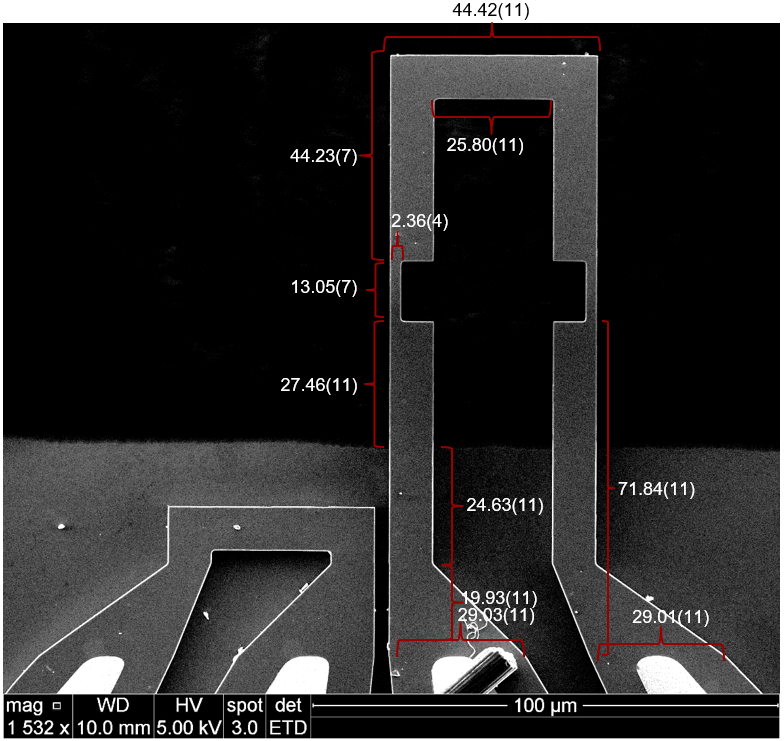


**Figure S6.** SEM image of microheater and dimensions measured in units of micrometers.


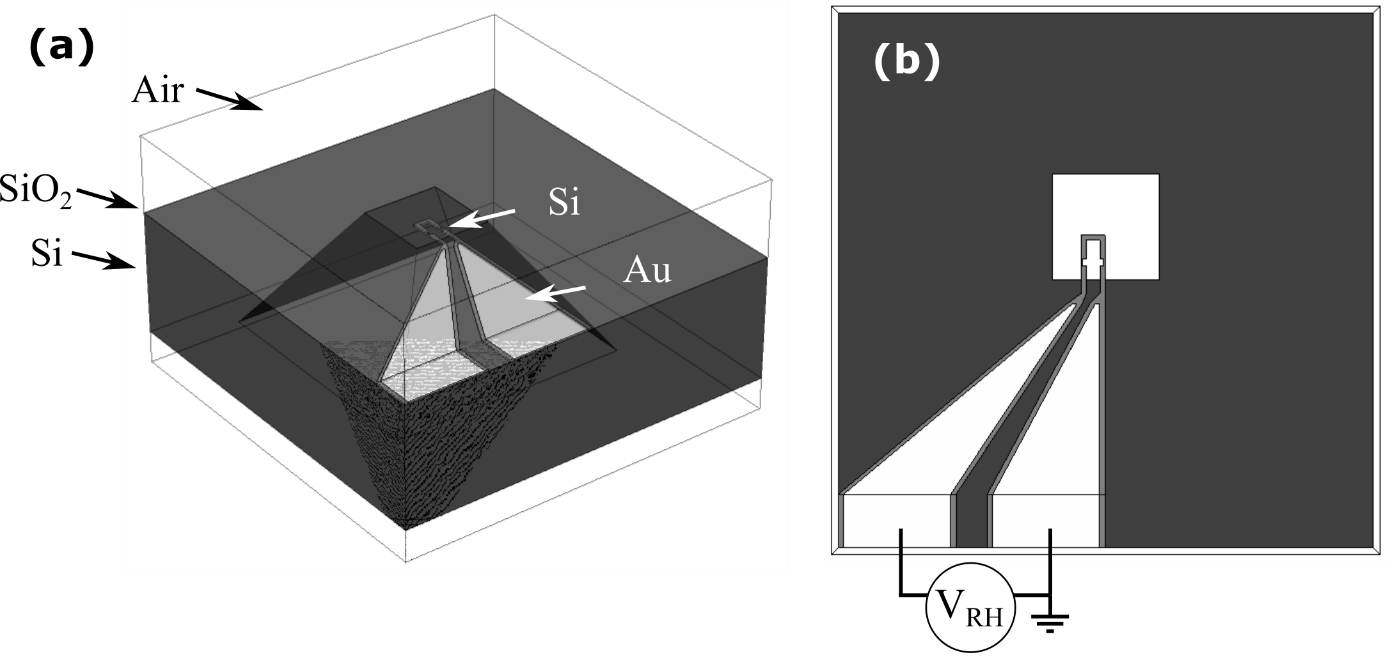


**Figure S7.** COMSOL model with materials indicated from topside (a) and from the top (b).

The physical properties were the standard properties in COMSOL except for the doping, which was found from electrical 4-point characterization of such devices to be (1.78 ± 0.12) x 10^17^ cm^-3^. The Arora mobility model was included to simulate the conductivity and carrier mobility at elevated temperatures.^[14]^ The thermal expansion coefficient for Si was expressed as a function of temperature^[15]^ as well as the thermal conductivity and heat capacity^[16]^. The thermal conductivity of air was added to the atmosphere as a function of temperature and pressure, which practically means, the thermal conductivity did not contribute to the negligible heat transfer when at vacuum conditions in the model. The boundaries of the chip base and microheater were fixed using the Solid Mechanics Module in COMSOL. The air domain was defined as a Fluid for the Heat Transfer Module to include its heat transfer effect. Black body radiation for heat transfer from the chip base was modelled with an emissivity of 0.68 and for the cantilever microstructure, it was set to 1 x 10^4^ as suggested in literature.^[11]^ For the Electric Current Module, a ground was chosen at one end of the Au-leads and the input terminal bias, *V*_RH_, at the other end of the microheater loop as indicated in **Figure S8**. The initial temperature was 25^o^C, while pressure was 1 x 10^-5^ Pa for vacuum conditions and 1 x 10^5^ Pa for air conditions. The smallest sizes of the mesh were 1 μm and the largest parts were 100 μm.


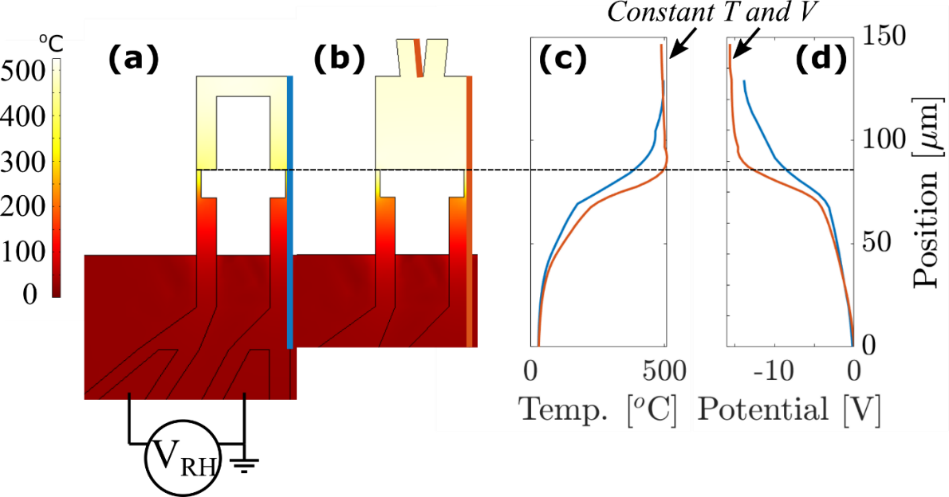


**Figure S8.** The temperature distribution in air has been compared for two different microheater designs: (a) The standard design and (b) a full cantilever with ”ears” at the maximum resistance peaks at 30.5 V and 31.5 V, respectively. The temperature (c) and potential (d) along the microheater sidewall are also indicated with an arrow indicating the more uniform temperature in the fully filled tip cantilever and ‘ear’ on it.

## 3.3 By Catalyst Melting Point

The temperature calibrations of the microheaters ex situ with Raman spectroscopy and simulations are time-consuming, as they demand many precise measurements of the chip geometry and material properties and has implicit assumptions. The calibrations also depend on the stability of the resistance where variations in contact resistance during experiments may lead to erroneous estimates of temperature if only relying on electrical measurements.

An attempt to find more stable and local temperature calibrations was made in situ during experiments in the ETEM. This was done by using the melting point of the Au-catalyst particles on the Si-microheaters and by analyzing the droplet composition of the catalyst for the GaAs nanowires. Au-nanoparticles were used as catalysts for the nanowire growth. When the Si-microheaters are heated by resistive heating, the nanoparticles will form liquid AuSi-alloys at a characteristic eutectic temperature, which is 363 ± 3^o^C for AuSi.^[17]^ The melting point can only be found at increasing temperatures as supercooling can hinder the formation of a solid at a well-defined decreasing temperature. The melting point can be found by looking at the contrast change of the particle going from a lighter contrast from the crystal lattice to a darker contrast for the dense liquid alloy (**Figure S9**). For a high-resolution TEM, it can also be revealed by the crystal lattice disappearing. The melting point can also be found by looking at the diffraction pattern of the Au-particles. The effect of other precursors should be avoided by doing the calibration in vacuum, before adding any precursors. It relies on a pure Si substrate, achieved by vapor HF etching the cantilever chip shortly before using in TEM.


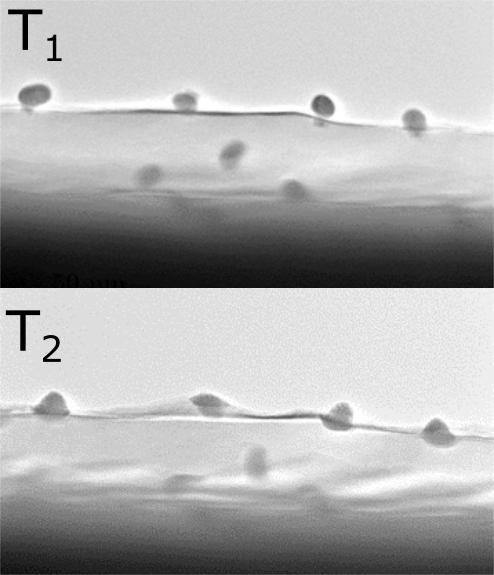


**Figure S9.** TEM images of Au-nanoparticles on top of Si-microheater sidewall being solid at T_1_ and forming liquid AuSi-eutectic alloy at T_2_ above the eutectic melting point being 363 ± 3^o^C.

The melting point was found to be at 27.75 ± 0.25 V by looking at the contrast change for one of the chips. Once the bias for the melting point is known, the temperature at higher biases can be found using linear extrapolation with the T vs V from the ex situ calibration. Both the resistance curve and the temperature curve are known to have linear segments before and after the peak resistance of the Si-cantilevers.^[18]^ These segments are illustrated in **Figure S10a** in region I and II, respectively. The linear coefficient of the temperature is 29 ± 2^o^C/V for region I and 54 ± 24^o^C/V for region II from the ex situ calibration in air. Similar coefficients are found from the simulations and calibration of another microheater from Raman Spectroscopy in air. The eutectic point is located within region I. Extrapolating the temperature to the resistance peak leads to a temperature of 487 ± 9^o^C (**Figure S10b**), which is approximately 25^o^C lower than the estimate from the simulation and almost 40^o^C lower than the ex situ calibration at the microheater tip, which is considered reasonable as the particle used for calibration here was also located at the microheater sidewall some micrometers further down the sidewall, where the temperature is expected to be lower according to the simulations. Determining the eutectic point is time consuming and the exact transition is hard to determine as the droplet starts to reshape at temperatures lower than the eutectic point. Besides the challenges of determining the melting point correctly, the sample may also be affected by impurities such as oxides or carbon deposition from the electron beam, which might prevent the AuSi-alloy from forming at the exact temperature of the melting point. This makes the method less attractive, however the great advantage is that temperature can be calibrated locally, when the AuSi-contact and microscope resolution are not the limiting factors.

## 3.4 By Droplet Composition

Temperature can also be determined from the Ga-concentration of the nanowire catalyst, once nanowires are growing.^[19,20]^ The catalyst consists primarily of Au and Ga as As has a low solubility in the catalyst. The amount of As is typically lower than 3% and can hardly be measured by EDX.^[20]^ The Ga-concentration depends also on the atmosphere e.g. V/III precursor ratio and pressure as well as contamination from other compounds such as silicon or indium. Two examples of the temperature as a function of composition are shown in **Figure S10c** and concentrations from droplets at similar conditions can be used to estimate the temperature. These reports are from the same ETEM as used in the present work, but the heating chips are commercial Norcada chips.^[21]^

**
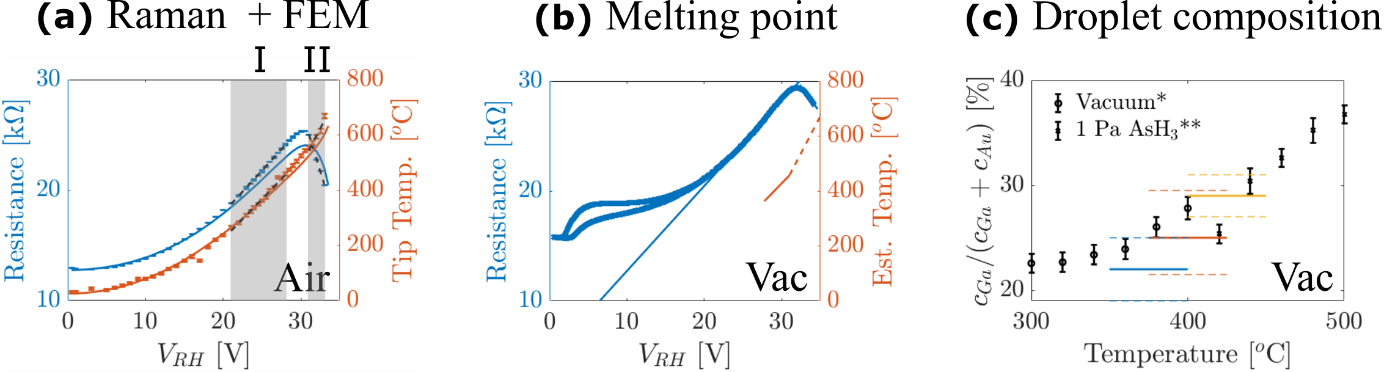
**

**Figure S10.** Resulting temperature calibration using Raman spectroscopy (a), AuSi-eutectic melting point and linear interpolation (b) and droplet composition (c). The estimated temperature for the droplet composition is at vacuum and 1 Pa AsH_3_ reported from previous work.^[19,20]^

## 3.5 Summary on temperature calibration methods

The temperature for different cantilever designs has previously been calibrated experimentally by different methods such as InfraRed Microscope images^[18]^, combining Raman Spectroscopy and experiments in COMSOL Multiphysics using a four-point design^[11]^ and using the growth parameters of Si-nanowires^[22]^. The four-point design was not available in the chips for the present work. The complexity of III-V materials does not allow using the growth rate of the nanostructures for temperature calibration as when growing Si NW. Therefore, it is necessary to consider other temperature characterization methods and their accuracy.

Temperature calibration is essential for the nucleation of nanowires, and it is not possible to do growth studies without nucleation. Raman spectroscopy has been used for ex situ calibration of the temperature behavior in an air atmosphere giving a temperature of 524 ± 6^o^C at the peak resistance. This is expected to be an overestimate because of the laser inducing a higher carrier mobility for temperatures at and above the peak resistance. The resistance and temperature are compared with Finite Element Models (FEMs), which have been solved for both an air and a vacuum atmosphere (**Figure S11**). The temperatures at the peak resistance from the simulations are 510 ± 2^o^C for both environments.


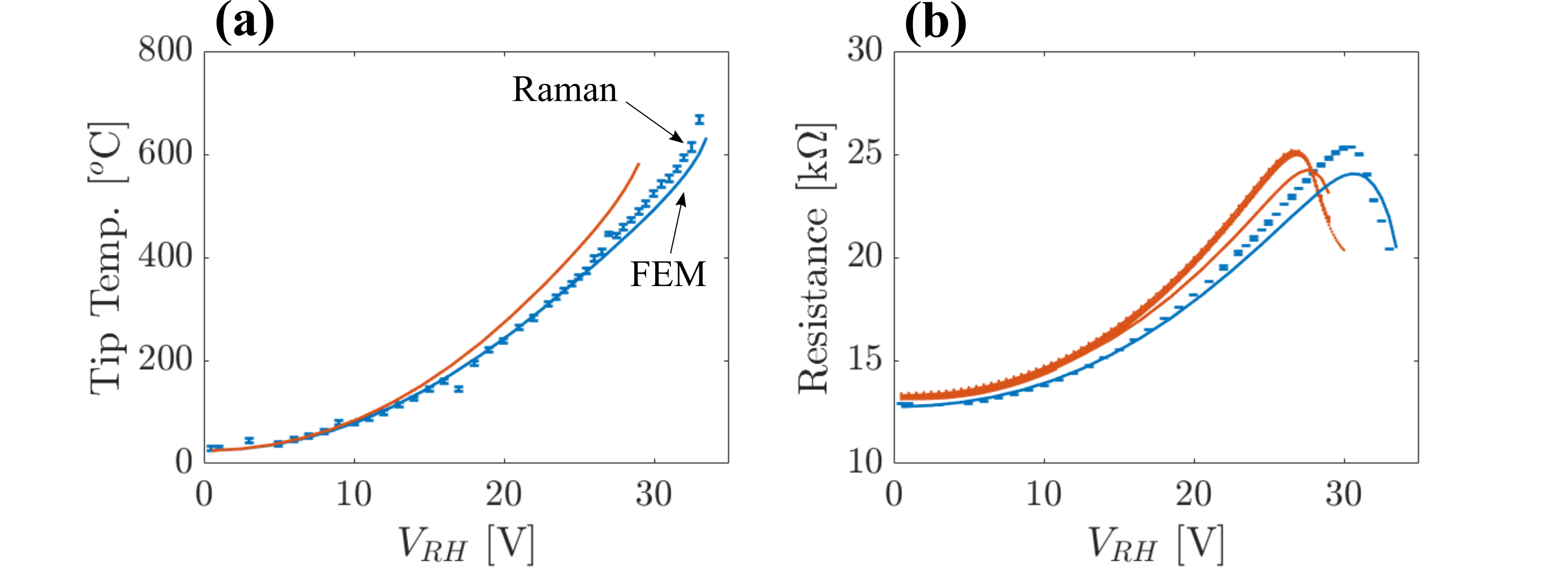


**Figure S11.** Temperature (a) and resistance (b) as function of applied bias (*V_RH_*) found from Raman spectroscopy and Finite Element Modelling (FEM) in an air atmosphere (blue) and vacuum (red).

The advantage of these Raman spectroscopy and simulations is that they can be used to find the temperatures from room temperature and up to 1000^o^C and validate the COMSOL FEM model. The disadvantages are that: 1) Each device should be calibrated to determine both geometrical parameters and the doping of the device. 2) These parameters may need further adjustment to make the simulation fit the experimental resistance and temperature. 3) The contact resistance leads to a difference in resistances, which should be corrected by adding the difference to the resistance from the simulations. 4) The resistance changes as bias is swept up and down, as there seems to be some changes in the gold layer on the leads, leading to lasting changes in resistance with time and use. 5) The resistance will change as material grows on the microheater sidewalls and changing atmospheres and illuminating the microheater with the electron beam in the microscope will change the resistance making temperature harder to interpret from the resistance and bias behavior.

Hence, in situ calibration methods are preferred over ex situ methods, as these are to some extent independent of the resistance behavior and microheater dimensions. They can also determine the temperature locally at the microheater, which is found from simulations to have a temperature gradient increasing 100^o^C along the microheater sidewall and 40^o^C from experimental results.

One in situ calibration method is the use of the melting point of the AuSi alloy at the microheater sidewall to determine temperature locally. However, the exact melting point can be hard to find from the contrast change and extrapolation of the temperature depends on ex situ calibration from either Raman spectroscopy or FEM. The melting point has been used in combination with linear extrapolation for a particle at the microheater sidewall suggesting a temperature of 487 ± 9^o^C at the position of the resistance peak. This is as expected for the particle at the sidewall a lower temperature than the estimated tip temperature from the ex situ calibration being 524 ± 9^o^C and 510 ± 2^o^C from simulations. Based on the results from the ex situ calibration methods and considering the effect of the position of the nanowire, the temperature at the peak resistance is estimated to be 500 ± 25^o^C at the microheater sidewall. The linear coefficients were 29 ± 2^o^C/V for the linear region before and 54 ± 24^o^C/V after the resistance peak.

Another way to determine the local temperature in situ is once nanowires grow; the Ga-concentration of the catalyst particle can be used to estimate the nanowire temperature with an estimated precision of 25^o^C, when comparing with pressure conditions similar to those reported in literature being either an AsH_3_ or vacuum. This adds some spatial resolution to the temperature calibration and is independent of the microheater parameters, which makes this method attractive.

Other ex situ methods for future temperature calibrations could be infrared imaging^[18]^, measuring Black Body radiation with an UV-Vis Spectrometer^[23]^ or Raman in a high vacuum atmosphere, which has not been possible to achieve with the equipment available for this work. Another possible in situ method is using selective area electron diffraction and beam parallelity to measure linear lattice expansion as a function of temperature.^[24]^ The expansion of the lattice parameters of e.g. the Si-microheaters or the GaAs nanowires at elevated temperatures can be compared with a reference from a commercial chip with well-known temperatures. This can also be used to measure the temperature gradient along the microheaters. ^[24]^ This method using diffraction to determine lattice expansion. Unfortunately, the diffraction spots shifted less than a pixel and the single crystals gave too few diffraction spots for the analysis. More accurate diffraction measurements should be acquired to make this calibration method work.

# Calculations of temperature change

The time, it takes to change temperature for the microheaters, can be calculated considering a simple physical system as described in the following.

The heat, *Q_e_*, can be calculated from the continuity equation and the resistive heating as

$C_{v}\frac{\partial T}{\partial t}-\nabla\left( k\nabla T \right)=Q_{e}=JE=\frac{\rho I^{2}}{({wh)}^{2}}$ (S12)

With *C_v_* being the volumetric heat capacity for the doped silicon, *T* being temperature, *k* being the thermal conductivity, *w* and *h* being the width and height, *ρ* being the resistance and *I* being the applied current initially.

At the hottest spot, $x=\frac{P}{2}$, with *P* being the perimeter of the loop, $\nabla\left( k\nabla T \right)=0$, which means:

$\frac{\partial T}{\partial t}=\frac{\rho I^{2}}{C_{v}({wh)}^{2}}$ (S13)

The solution of the differential equation is

$T\left( t \right)=T_{0}+\frac{\rho I^{2}}{C_{v}({wh)}^{2}}t$ (S14)

The time given in **Equation S14** is found by isolating *t*.

Turning off power, the time it takes to cool down with the chip as heat reservoir is found by:

$\frac{dT(t)}{dt}=-\frac{1}{\tau}(T-T_{0})$ (S15)

With

$\tau=\frac{mC_{v}P}{whk}=\frac{C_{v}P^{2}}{k}$ (S16)

The solution of the differential equation is

$T\left( t \right)=T_{0}+\left( T-T_{0} \right)e^{-t/\tau}$ (S17)

The time given in **Equation S17** is found by isolating *t*.

# Crystal phase categorization

The stable bulk phase of most III-V materials is the cubic zincblende (ZB). While a high surface-to-volume ratio at the nanometer scale means surface effects cannot be ignored allowing the formation of the meta-stable hexagonal wurtzite (WZ) structure not present in the bulk.^[25,26]^ These two phases are typically distinguished by the stacking sequence of the close packed planes. ZB consists of crystal layers of the diatomic binding of group III and group V with three different sequential positions …ABCABC…, while WZ only takes two different positions …ABABAB…. The positions of the atoms of a single bilayer may differ from the rest of the sequence. This is known as stacking faults for WZ, when for instance an erroneous bilayer is formed interrupting the bilayer sequence, or as a twin plane in ZB, mirroring the sequence before and after a misplaced bilayer.

The crystal layers have been characterized by analyzing TEM images of nanowires at the specific conditions. The characterization of the crystal phase has been made by cropping a region of interest and summing the intensity of the pixels at the nanowire width. An example of a characterization is illustrated in **Figure S18**. The intensity peaks indicate the crystal layers and behavior of multiple peaks can be used to define the crystal phases. Zincblende (ZB) has clear individual peaks for each monolayer, while wurtzite (WZ) has two characteristic peaks for each monolayer in a bilayer. Stacking faults (SF) are anomalous in the periodicity of the two types of peaks.


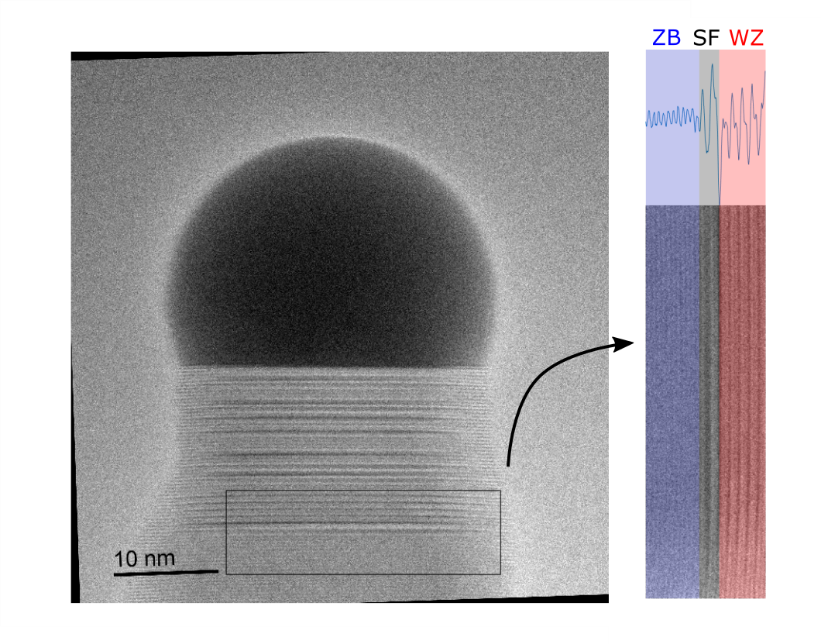


**Figure S18.** Illustration of a transition from ZB to WZ with a SF at the transition. This is an expansion of the inset in Figure 1d.

# Crystal phase map

The overall aim of the crystal phase map in **Section 2.1**, from which both the example of **Figure 2d** and **Figure 3b** originate, is to determine the conditions with either crystal phase, WZ or ZB, dominate to find the optimal conditions forming phase-pure structures. This is used and confirmed for nanowires with different lengths in the study of the temperature induced crystal phase change (**Section 2.3**) and exemplified by the initial nanowire shown in **Figure 5** and the top segment of the nanowire in **Figure S23** with phase-pure WZ. Similarly, phase-pure ZB was observed at low temperatures when mapping the crystal phases. At these low temperatures ZB was the only phase forming as mentioned in the main text and **Table S19**. However, it is known that the electron beam may induce stacking faults as reported elsewhere^[27]^ and the low pressure growth is sensitive fluctuations in the precursor flow.

**Table S19.** Distribution of crystal monolayers at specific [V]/[III]-ratios and temperatures. Each entry quantifies the count of Wurtzite (WZ), Zincblende (ZB), and Stacking Fault (SF) monolayers, color-coded in red, blue, and black, respectively, at the specific growth conditions. The distribution was counted at a constant [V]/[III]-ratio with corresponding partial pressures of TMGa over AsH_3_ noted in parenthesis and a constant bias with corresponding estimated temperatures noted in parenthesis.

| [V]/[III]-ratio (*p*AsH_3_/*p*TMGa)/Voltage (Temp.) | 26.5 V (420^o^C) | 27.75 V (450^o^C) | 29 V (495^o^C) |
| --- | --- | --- | --- |
| 224 (0.13 Pa / 0.57 x 10^-3^ Pa) |  | 0/29/0 | 0/77/0 |
| 448 (0.34 Pa / 0.75 x 10^-3^ Pa) |  | 0/14/2 | 0/17/0 |
| 673 (0.93 Pa / 1.39 x 10^-3^ Pa) |  | 15/0/0 |  |
| 897 (1.19 Pa / 1.33 x 10^-3^ Pa) | 0/14/2 | 10/10/2 | 34/0/0 |
| 1345 (1.67 Pa / 1.24 x 10^-3^ Pa) | 0/42/3 |  |  |

# Droplet Volume and Contact Angles

The contact angle, the angle between the droplet-nanowire interface and the droplet surface, is a geometrical parameter, which can easily be measured from the projected droplet-nanowire during in situ measurement (**Figure S20a-b**). In our study, we determined a characteristic contact angle of approximately 107^o^, where WZ was forming at lower contact angles, while ZB was dominating at higher contact angles (**Figure S20a**). Although conceptually in agreement with other in situ studies showing a cutoff-angle between WZ and ZB, our observed value is below the one of ≈125^o^ reported earlier. Potential explanations could be differences in the growth conditions, the fact that the actual determining parameters such as the droplet volume are hardly transferred to its projection as contact angle (**Figure S20c**) or unavoidable contamination in the system as elaborated in the next section.


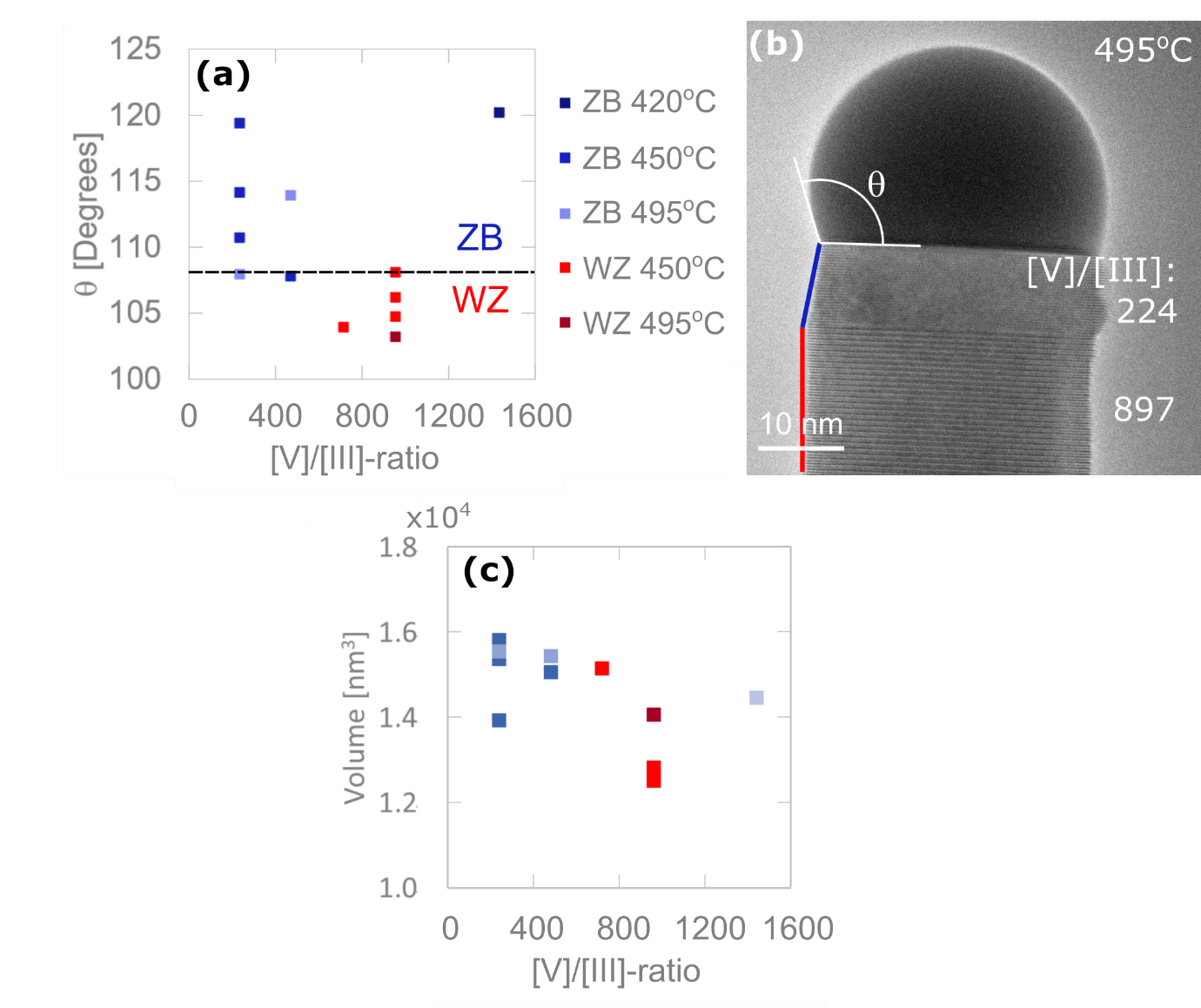


**Figure S20.** (a) Crystal Phase diagram of contact angles at different [V]/[III]-ratios and temperatures. (b) A micrograph illustrates how contact angle has been defined. (c) Crystal Phase diagram of volume at different [V]/[III]-ratios and temperatures.

The analysis of the contact angle and volume are also reported for the change in pressure and temperature (**Figure S21**) and for a single crystal phase quantum dot formation (**Figure S22**) to illustrate their behavior further.


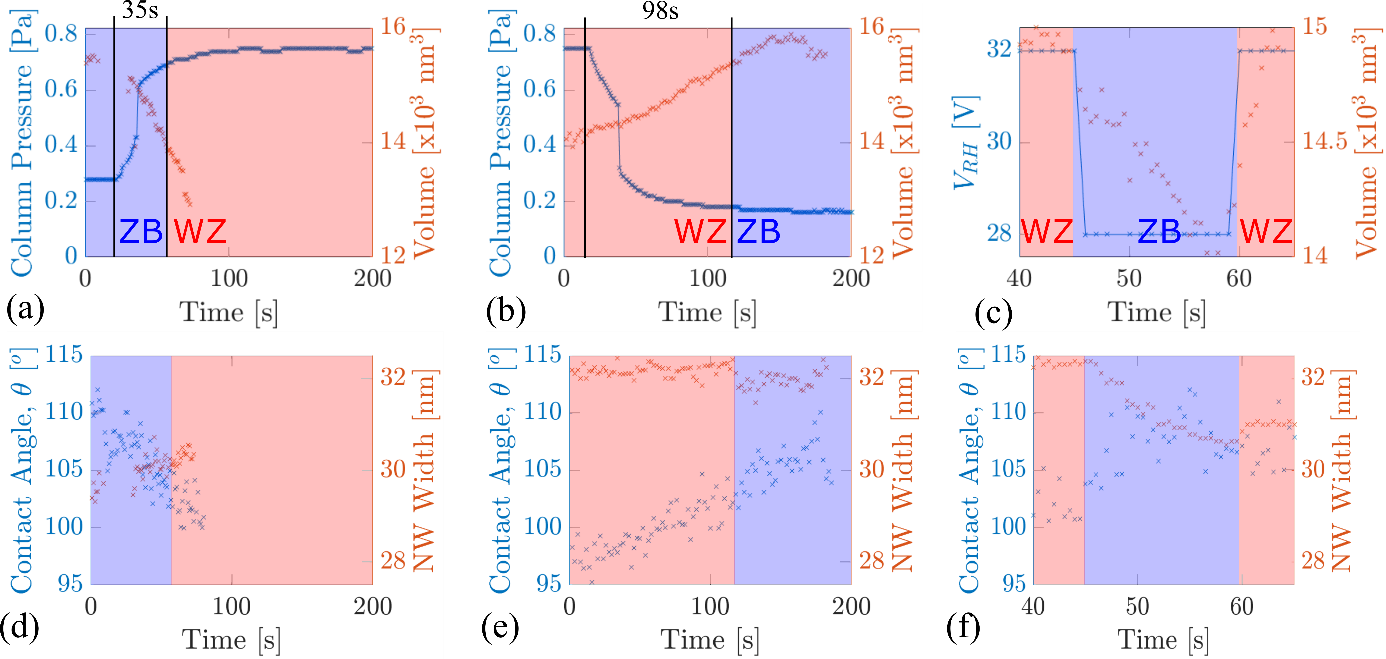


**Figure S21.** Volume, nanowire (NW) width and contact angle for the time dependent transitions illustrated for (a,b,d,e) Figure 3 and (c,f) figure 4 in the main text.


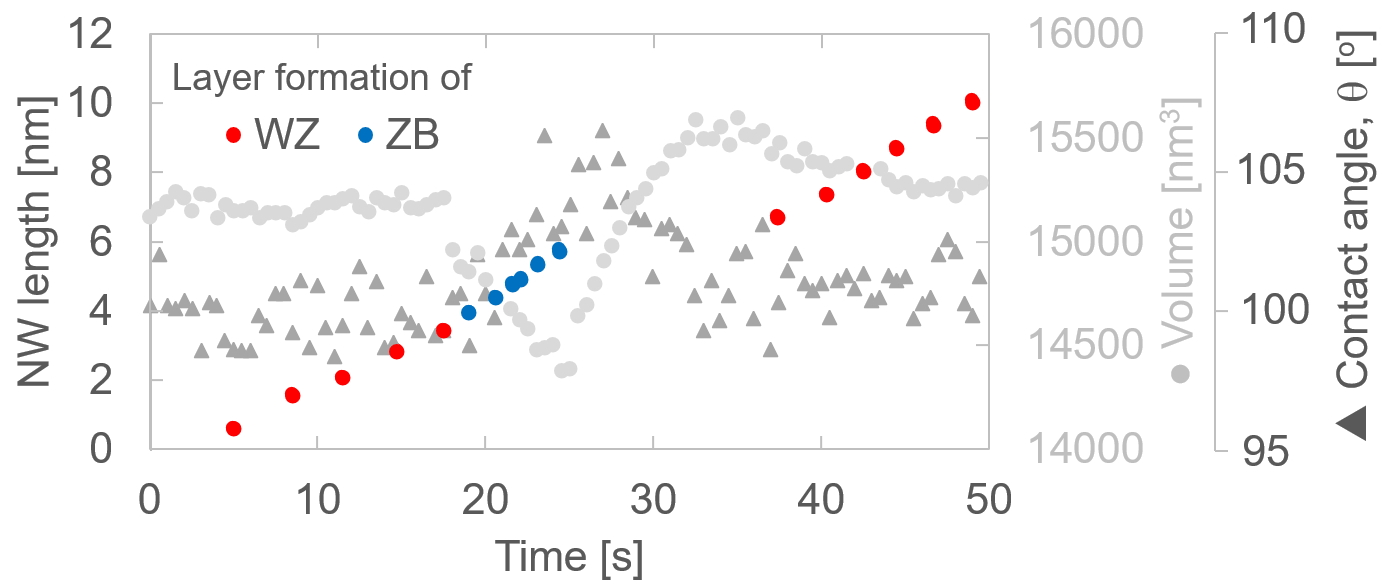


**Figure S22.** Volume and contact angle for the time dependent transition illustrated for Figure 5c in the main text.

# Crystal Phase Quantum Dots

The individual quantum dots reported in **Figure 5** in the main text are shown in **Figure S23**.

The study was made at three different time spans during the same experiment in a single nanowire. The high temperature forming wurtzite was increased with approximately 15^o^C each time from the first session (**Figure S23a**) to the second session (**Figure S23b**) and further for the third session with (**Figure S23c**) (See the different bias applied to the microheaters in **Table S26-27**). This resulted in increasing radial growth as temperature was increased as seen for the nanowires in the three overviews.


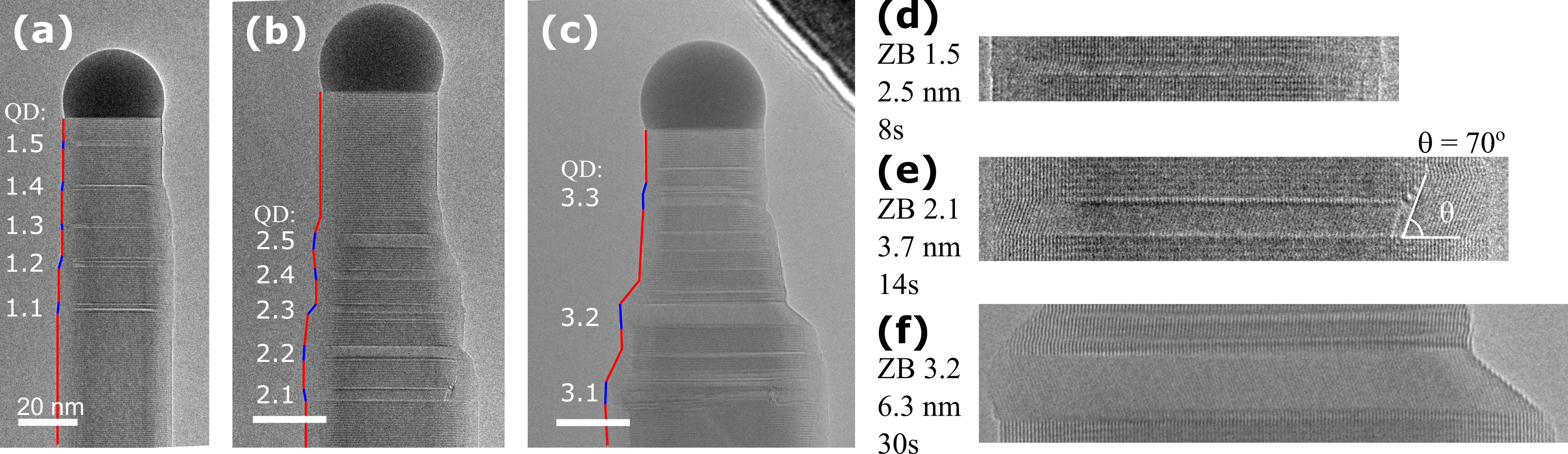


**Figure S23.** The nanowires with different segments reported in the main text grown at different time steps. Examples of different sized segments are shown in (d)-(f). Vertical twins grown close to the sidewall of the nanowire are shown for segment 2.1 in (e). This has an angle of 70^o^. Similar behavior has been seen in previous reports.^[28]^


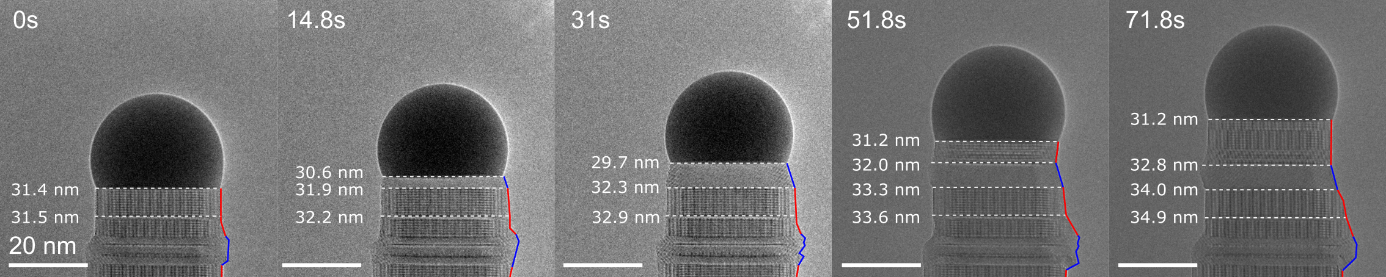


**Figure S24**. Movie sequence forming segment QD3.2 from Figure S23c. The diameter of different WZ and ZB segments are illustrated and increases over time as indicated.


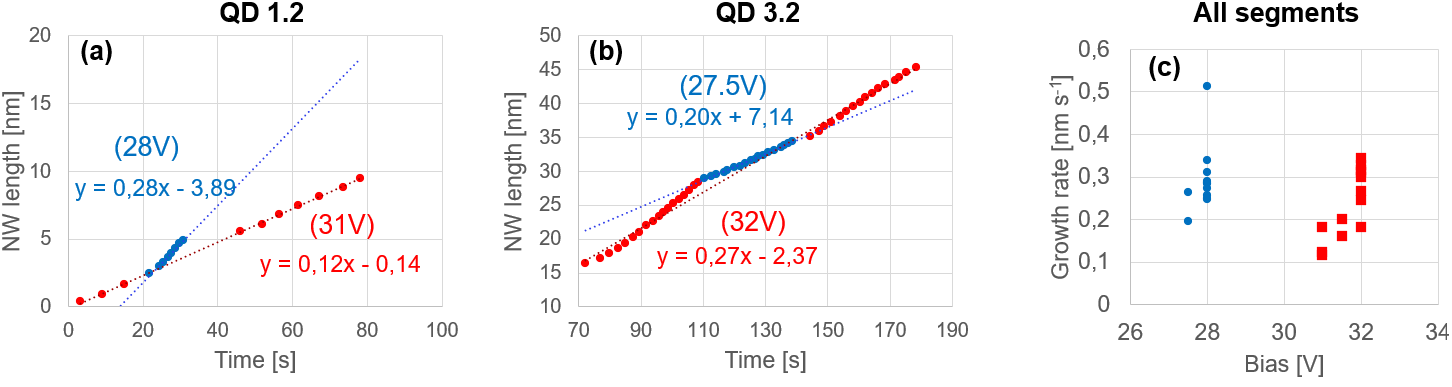


**Figure S25.** The growth rate for different temperatures and segments. Examples of (a) QD1.2 and (b) QD3.2 with WZ and ZB segments grown at different temperatures and an estimate of their growth rates found from linear trend lines.

The time of incubation and growth steps forming WZ and ZB are given in **Table S26** and **S27**, respectively. This is the average step when forming a bilayer or a single layer, respectively. The reported time is worth comparing with the time it takes to change temperature, which is less than a millisecond as discussed in the main text.

**Table S26.** Time of incubation and growth step forming WZ at different temperatures.

| Bias | incubation | Step | Observations |
| --- | --- | --- | --- |
| 31V | 3.9 ± 1.0 s | 1.6 ± 0.7 s | 30 layers |
| 31.5V | 1.5 ± 0.5 s | 2.3 ± 0.5 s | 22 layers |
| 32V | 1.20 ± 0.4 s | 1.0 ± 0.4 s | 229 layers |

**Table S27.** Time of incubation and growth step forming ZB at different temperatures.

| Bias | Incubation | Step | Observations |
| --- | --- | --- | --- |
| 28V | 0.4 ± 0.2 s | 0.9 ± 0.9 s | 98 layers |
| 27.5V | 0.7 ± 0.1 s | 0.9 ± 0.5 s | 40 layers |

# Droplet and nanowire composition

An overview of the droplet and nanowire composition at different growth conditions for the phase diagram reported in **Figure 2a** (**Table S28**) and one of the nanowires forming the crystal phase quantum dots reported in **Figure 5** (**Table S29**). Cross contamination from other experiments in the ETEM left trace amounts of In within droplets assisting GaAs nanowire growth. However, this have been shown to have minimal effect on the nanowire composition^[29]^, which is supported by the similarities between the observed crystal phase trends of **Figure 2a** in the main text and previous reports using the same setup for GaAs^[20]^. Hence, the contamination of the droplet does not seem to be affecting the global tendencies of the growth parameter-dependent crystal phase formation reported here.

**Table S28.** Overview of droplet composition at different temperatures and [V]/[III]-ratios used to map the crystal phase diagram in the main text.

| Temp. | [V]/[III]-ratio | Ga [%] | As [%] | In [%] | Au [%] | Droplet Volume [nm^3^] |
| --- | --- | --- | --- | --- | --- | --- |
| 422^o^C | 1345 | 28 ± 5 | 2 ± 3 | 0 ± 3 | 69 ± 2 | 14463 ± 38 |
| 451^o^C | 224 | 30 ± 4 | 2 ± 2 | 8 ± 2 | 60 ± 2 | 15048 ± 979 |
| 451^o^C | 897 | 12 ± 3 | 2 ± 2 | 10 ± 2 | 78 ± 2 | 12656 ± 159 |
| 494^o^C | 224 | 48 ± 8 | 3 ± 3 | 2 ± 3 | 47 ± 3 | 15552 ± 78 |
| 494^o^C | 897 | 23 ± 4 | 0 ± 2 | 5 ± 2 | 72 ± 2 | 14071 ± 39 |

**Table S29.** Overview of droplet and nanowire composition used for the segment formation reported in the main text. Micrograph indicates the region used for EDX measurements.

| 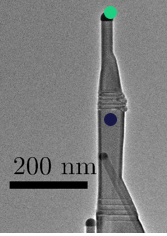 | Ga [%] | As [%] | In [%] | Au [%] |
| --- | --- | --- | --- | --- |
| Droplet | 15.5 ± 1.3 | 3.0 ± 0.9 | 22.2 ± 1.4 | 59.4 ± 1.0 |
| Nanowire | 47.9 ± 1.0 | 51.3 ± 0.9 | 0.9 ± 0.2 | 0 |

# References

[1] J. B. Cui, K. Amtmann, J. Ristein, L. Ley, *J Appl Phys* **1998**, *83*, 7929.

[2] G. Viera, S. Huet, L. Boufendi, *J Appl Phys* **2001**, *90*, 4175.

[3] R. Ostermeir, K. Brunner, G. Abstreiter, W. Weber, in *European Solid-State Device Research Conference*, IEEE Computer Society, **1990**, pp. 591–4.

[4] S. Périchon, V. Lysenko, B. Remaki, D. Barbier, B. Champagnon, *J Appl Phys* **1999**, *86*, 4700.

[5] H. Brugger, P. W. Epperlein, *Appl Phys Lett* **1990**, *56*, 1049.

[6] P. W. Epperlein, G. L. Bona, P. Roentgen, *Appl Phys Lett* **1992**, *60*, 680.

[7] M. Kuball, S. Rajasingam, A. Sarua, M. J. Uren, T. Martin, B. T. Hughes, K. P. Hilton, R. S. Balmer, *Appl Phys Lett* **2003**, *82*, 124.

[8] A. Chitnis, J. Sun, V. Mandavilli, R. Pachipulusu, S. Wu, M. Gaevski, V. Adivarahan, J. P. Zhang, M. A. Khan, A. Sarua, M. Kuball, *Appl Phys Lett* **2002**, *81*, 3491.

[9] M. R. Abel, T. L. Wright, W. P. King, S. Graham, *IEEE Transactions on Components and Packaging Technologies* **2007**, *30*, 200.

[10] S. P. Kearney, L. M. Phinney, M. S. Baker, *Journal of Microelectromechanical Systems* **2006**, *15*, 314.

[11] D. S. Engstrøm, N. L. Rupesinghe, K. B. K. Teo, W. I. Milne, P. Bøgild, *Journal of Micromechanics and Microengineering* **2011**, *21*, 015004.

[12] M. Gan, V. Tomar, *Review of Scientific Instruments* **2014**, *85*, 013902.

[13] COMSOL Multiphysics® V. 6.0 www.comsol.com. COMSOL AB, Stockholm Sweden.

[14] N. D. Arora, J. R. Houser, D. J. Roulston, *IEEE Trans Electron Devices* **1982**, *ED-29*, 292.

[15] C. A. Swenson, *J Phys Chem Ref Data* **1983**, *12*, 179.

[16] V. Košel, R. Sleik, M. Glavanovics, in *Collection of Papers Presented at The 13th International Workshop on THERMal INvestigation of ICs and Systems, THERMINIC*, **2007**, pp. 110–4.

[17] H. Okamoto, T. B. Massalski, *Bulletin of Alloy Phase Diagrams* **1983**, *4*, 810.

[18] J. Lee, T. Beechem, T. L. Wright, B. A. Nelson, S. Graham, W. P. King, *Journal of Microelectromechanical Systems* **2006**, *15*, 1644.

[19] M. Tornberg, D. Jacobsson, A. R. Persson, R. Wallenberg, K. A. Dick, S. Kodambaka, *Nano Lett* **2019**, *19*, 3498.

[20] C. B. Maliakkal, D. Jacobsson, M. Tornberg, A. R. Persson, J. Johansson, R. Wallenberg, K. A. Dick, *Nat Commun* **2019**, *10*, 4577.

[21] J. Y. Howe, M. S. Thompson, S. Dogel, K. Ueda, T. Matsumoto, H. Kikuchi, M. Reynolds, H. Hosseinkhannazer, T. J. Zega, *Microscopy and Microanalysis* **2017**, *23*, 66.

[22] C. Kallesøe, C. Y. Wen, K. Mølhave, P. Bøggild, F. M. Ross, *Small* **2010**, *6*, 2058.

[23] P. W. Atkins, J. De Paula, *Physical Chemistry*, W.H. Freeman, New York, USA **2006**.

[24] F. Niekiel, S. M. Kraschewski, J. Müller, B. Butz, E. Spiecker, *Ultramicroscopy* **2017**, *176*, 161.

[25] M. Koguchi, H. Kakibayashi, M. Yazawa, K. Hiruma, T. Katsuyama, *Jpn J Appl Phys* **1992**, *31*, 2061.

[26] F. Glas, J. C. Harmand, G. Patriarche, *Phys Rev Lett* **2007**, *99*, 146101.

[27] M. Tornberg, C. B. Maliakkal, D. Jacobsson, R. Wallenberg, K. A. Dick, *Microscopy and Microanalysis* **2022**, *28,* 1484.

[28] S. Lehmann, D. Jacobsson, K. Deppert, K. A. Dick, *Nano Res* **2012**, *5*, 470.

[29] R. Sjökvist, D. Jacobsson, M. Tornberg, R. Wallenberg, E. D. Leshchenko, J. Johansson, K. A. Dick, *Journal of Physical Chemistry Letters* **2021**, *12*, 7590.
